# B-Cyclin/CDKs Regulate Mitotic Spindle Assembly by Phosphorylating Kinesins-5 in Budding Yeast

**DOI:** 10.1371/journal.pgen.1000935

**Published:** 2010-05-06

**Authors:** Mark K. Chee, Steven B. Haase

**Affiliations:** Department of Biology, Duke University, Durham, North Carolina, United States of America; Fred Hutchinson Cancer Research Center, United States of America

## Abstract

Although it has been known for many years that B-cyclin/CDK complexes regulate the assembly of the mitotic spindle and entry into mitosis, the full complement of relevant CDK targets has not been identified. It has previously been shown in a variety of model systems that B-type cyclin/CDK complexes, kinesin-5 motors, and the SCF^Cdc4^ ubiquitin ligase are required for the separation of spindle poles and assembly of a bipolar spindle. It has been suggested that, in budding yeast, B-type cyclin/CDK (Clb/Cdc28) complexes promote spindle pole separation by inhibiting the degradation of the kinesins-5 Kip1 and Cin8 by the anaphase-promoting complex (APC^Cdh1^). We have determined, however, that the Kip1 and Cin8 proteins are present at wild-type levels in the absence of Clb/Cdc28 kinase activity. Here, we show that Kip1 and Cin8 are *in vitro* targets of Clb2/Cdc28 and that the mutation of conserved CDK phosphorylation sites on Kip1 inhibits spindle pole separation without affecting the protein's *in vivo* localization or abundance. Mass spectrometry analysis confirms that two CDK sites in the tail domain of Kip1 are phosphorylated *in vivo*. In addition, we have determined that Sic1, a Clb/Cdc28-specific inhibitor, is the SCF^Cdc4^ target that inhibits spindle pole separation in cells lacking functional Cdc4. Based on these findings, we propose that Clb/Cdc28 drives spindle pole separation by direct phosphorylation of kinesin-5 motors.

## Author Summary

The assembly of a bipolar mitotic spindle is essential for the accurate segregation of sister chromatids during mitosis and, hence, for successful cell division. Spindle assembly depends on the successful duplication of the spindle poles, followed by their separation to opposing ends of the cell. Although it has been known for many years that B-cyclin/CDK complexes regulate the assembly of the mitotic spindle, the relevant CDK targets have not been identified. Motor proteins of the kinesin-5 family generate movement on the microtubules that make up the spindle and are believed to power spindle pole separation. By employing the budding yeast *Saccharomyces cerevisiae* as a model, we have found evidence that cyclin/CDKs control spindle assembly by phosphorylating the kinesins-5 Kip1 and Cin8. When phosphorylation at a conserved CDK site in the motor domain of Kip1 is blocked, spindle pole separation is greatly diminished but neither protein abundance nor localization is affected. We have also obtained direct evidence by mass spectrometry that Kip1 and Cin8 are phosphorylated *in vivo* at consensus CDK sites in their tail domains. Our findings suggest that B-cyclin/CDKs regulate spindle assembly by regulating kinesin-5 motor activity.

## Introduction

Cyclin-dependent kinases (CDKs) complexed with various cyclins coordinate many duplication and segregation events during the eukaryotic cell division cycle [1], [2]. The duplication of the cell's microtubule organizing center, the centrosome, and the subsequent separation of the duplicated centrosomes is one such event [3], [4]. Timely separation of the duplicated centrosomes is required for the assembly of the bipolar spindle at metaphase which, in turn, is necessary for the equal segregation of sister chromatids during anaphase and the preservation of genome stability.

The budding yeast centrosome, called the spindle pole body (SPB), is functionally equivalent to the metazoan centrosome. Although structurally dissimilar [5], they appear to be regulated by similar mechanisms [3], [6]. Thus, the budding yeast SPB is a powerful model for understanding the metazoan centrosome, as demonstrated by genetic studies that have identified many components of the eukaryotic cellular machinery critical to both SPB and centrosome separation (reviewed in [5]–[9]).

Three classes of mutations that cause cells to arrest with duplicated but unseparated SPBs have been identified in *Saccharomyces cerevisiae*. The first class includes mutations in the genes encoding Cdc28, the yeast Cdk1, and the B-cyclins which bind to Cdc28. Cells lacking all six B-type cyclin genes (*CLB1*, *CLB2*, *CLB3*, *CLB4*, *CLB5*, and *CLB6*) are unable to separate SPBs [4]. The mutation of tyrosine 19 in Cdc28 to mimic an inhibitory phosphorylation (*cdc28^Y19E^*) [10], [11] has also been reported to result in a SPB separation defect [12]. This phosphomimetic mutation is thought to specifically inhibit Clb1,2,3,4/Cdc28 complexes, but likely not Clb5,6/Cdc28 [11]. Not surprisingly, Δ*clb1,2,3,4* mutants also appear to have a diminished capacity to separate SPBs [13], , although separation can occur after extended time periods [4], [13].

The second class of SPB separation mutations affects genes encoding components of the SCF^Cdc4^ E3 ubiquitin ligase complex (*CDC4*, *CDC53* and *SKP1* [15]–[17]) as well as *CDC34* [18], the E2 ubiquitin protein-conjugating enzyme that is associated with SCF^Cdc4^. Temperature-sensitive *cdc4*, *cdc53*, and *cdc34* mutants arrest with multiple elongated buds and unreplicated DNA, as well as duplicated but unseparated SPBs [15]–[19]. The arrest phenotype of these mutants is likely to be identical to that of Δ*clb1,2,3,4,5,6* [4], [20] mutants due to a buildup of Sic1 [15], [20]. Sic1 is a Clb/Cdc28-specific inhibitor whose degradation is normally triggered by the SCF^Cdc4^ complex in G1 to allow entry into S phase [20]–[22]. However, it is possible that there is a SCF^Cdc4^ target that is directly involved in maintaining cohesion between the duplicated SPBs and which must be destroyed before separation can occur. Such a protein could be a component of the proteinaceous bridge structure that physically joins newly duplicated SPBs and would need to be overcome for separation to occur [8], [9]. Direct phosphorylation by CDK complexes is generally required to trigger the ubiquitination of SCF targets [23]; so Clb/Cdc28 complexes might work in concert with the SCF^Cdc4^ to destroy such a separation-inhibiting element.

The third class of mutations lies in the *KIP1* and *CIN8* genes [24], [25] which encode members of the kinesin-5 family of bipolar, microtubule-based motor proteins [26]. Kinesins-5 have been shown to be important in both the establishment and maintenance of the bipolar spindle in many fungal and metazoan systems [27]–[30]. It is thought that kinesin-5 motors crosslink and move spindle microtubules, which are also required for SPB separation [31], [32], in order to mechanically separate the spindle poles and establish the spindle (reviewed in [33]). Accordingly, cells lacking both functional Kip1 and Cin8, arrest with duplicated and unseparated SPBs when released from a G1 arrest [24], [25].

Together, these findings suggest that Clb/Cdc28 complexes promote the timely separation of SPBs, and that kinesin-5 motors may be subject to phosphoregulation by Clb/Cdc28 complexes [14], [34]. Although several of the genetic requirements for SPB separation are now known, the molecular mechanisms that regulate separation remain unclear. cyclin B/Cdk1 phosphorylation of the tail domain of the *Homo sapiens* [35], [36], *Xenopus laevis* [37], [38], and *Drosophila melanogaster* [39], [40] kinesin-5 orthologues (HsEg5 or Kif11, XlEg5, and KLP61F, respectively) has been shown to be required for their localization to the spindle. The BimC box motif [28] where this phosphorylation occurs is not found, however, in either Kip1 or Cin8, although other consensus CDK phosphorylation sites exist in both proteins.

Two recent studies [41], [42] suggest that Clb/Cdc28 complexes regulate kinesin-5 protein stability indirectly by phosphorylating Cdh1, a substrate-specific activator of the anaphase-promoting complex (APC) [43]. The APC^Cdh1^ is active in G1, and is thought to be inactivated in S phase by B-cyclin/CDK-mediated phosphorylation of Cdh1 [44], [45]. The degradation of Kip1 and Cin8 is dependent on the APC in complex with Cdc20 [46] or with Cdh1 [47], respectively. Motor stability thus depends on APC activity. The studies by Crasta et al., however, relied heavily on two mutant alleles of *CDC28*, *cdc28^Y19E^* and *cdc28-as1*, which retain some CDK activity under the experimental conditions employed [41], [48]. They therefore do not rule out the possibility that CDKs also regulate kinesin-5 motors directly.

In this study, we asked if Clb/Cdc28 directly regulates kinesin-5 activity in order to trigger SPB separation and spindle assembly. We first determined that the only target of SCF^Cdc4^ involved in regulating SPB separation is the Clb/Cdc28-specific inhibitor, Sic1. Thus, SCF-mediated destruction of a bridge component is likely not required for spindle assembly. We next determined that Clb2/Cdc28 phosphorylates Kip1 and Cin8 *in vitro*, and also that Clb/Cdc28 complexes do not regulate either the abundance or localization of these kinesins-5 *in vivo*. Moreover, by genetic mapping, we identified a CDK phosphorylation site in the motor domain of Kip1 that is critical to SPB separation. We also identified two non-conserved CDK sites in the tail domain of Kip1 that are important for timely SPB separation, and verified that they are phosphorylated *in vivo* by mass spectrometry. As the site in the motor domain is conserved across almost all of the kinesin-5 family, we propose that direct regulation of kinesin-5 motor functions by B-cyclin/CDK may not be exclusive to *S. cerevisiae*.

## Results

### Deleting *SIC1* is sufficient to permit SPB separation in cells lacking functional Cdc4

Mutants lacking SCF^Cdc4^ E3 ubiquitin ligase activity fail to separate duplicated SPBs. This observation suggests that there is a protein or proteins that must be ubiquitinated by the SCF^Cdc4^ and subsequently degraded in order for the SPBs to separate. A likely candidate is the Clb/Cdc28-specific inhibitor Sic1, which accumulates at the restrictive temperature in mutants with temperature-sensitive alleles of the SCF^Cdc4^ complex components such as Cdc4, Cdc34, Cdc53, and Skp1 [15], [20]. Sic1 inhibits Clb/Cdc28 kinases essential for S-phase entry [15], [20] as well as SPB separation [4], [13]. Moreover, cells that express a hyperstabilized allele of Sic1 arrest with a phenotype identical to that of cells which lack SCF^Cdc4^ activity [49]. These findings do not, however, rule out the possibility that the destruction of additional SCF^Cdc4^ targets, such as components of the bridge structure which physically joins newly duplicated SPBs, may be essential for SPB separation.

**Figure 1:**
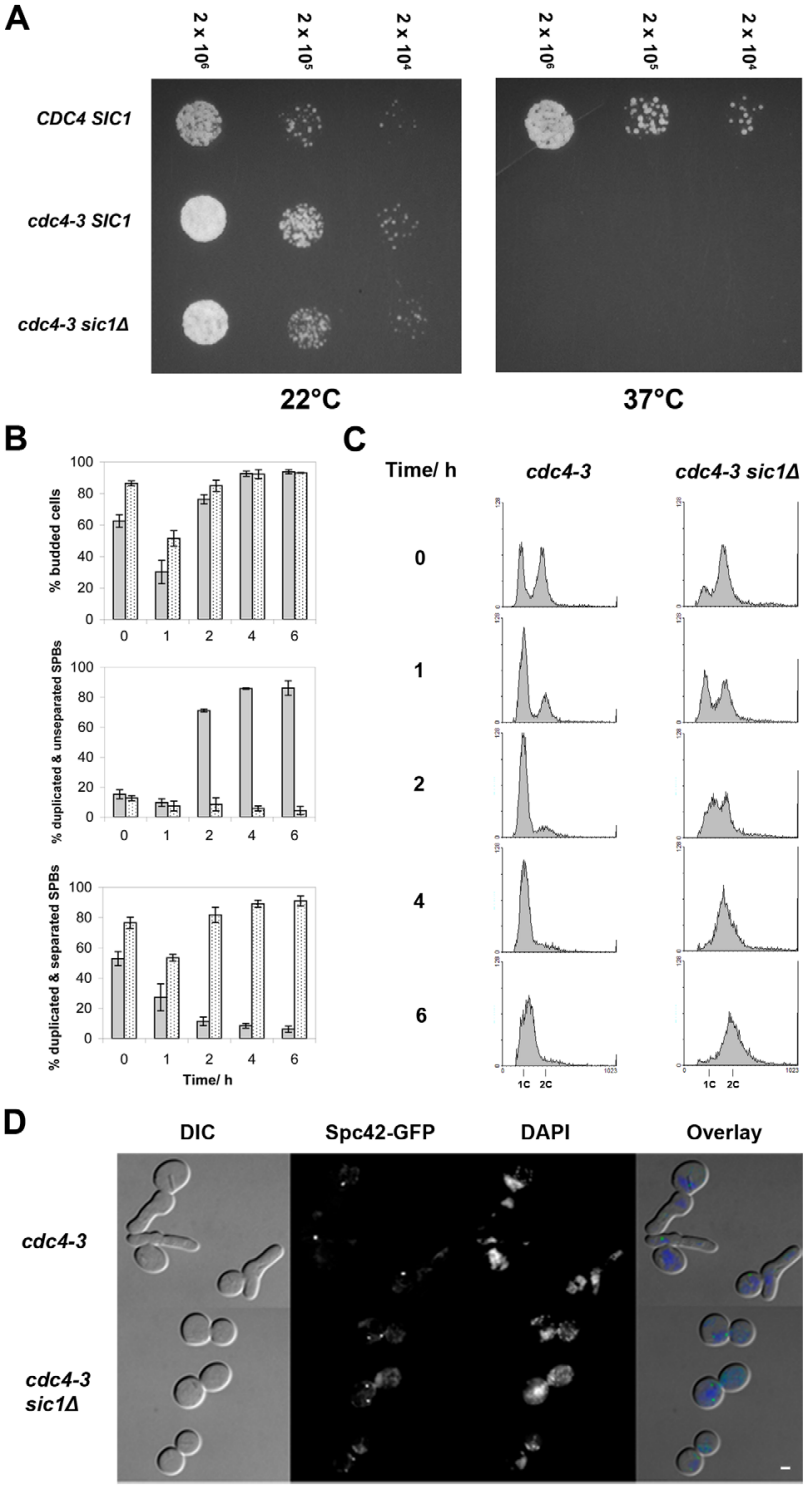
*SIC1* deletion allows SPB separation and DNA replication in *cdc4-3*(ts) cells at the restrictive temperature. (A) Spot assay showing that *cdc4-3 sic1*Δ cells arrest at 37°C. Strains growing in log phase at permissive temperature were diluted to 2×10^6^ cells/ml, and further diluted serially to 2×10^4^ cells/ml. An equal volume of cells from each dilution was spotted on YEPD, and plates were incubated at either ambient temperature (∼22°C) or 37°C. (B) Percentages of budded cells, cells with duplicated and separated SPBs, and cells with duplicated but unseparated SPBs are shown for asynchronous log phase cultures shifted from permissive (24°C) to restrictive (37°C) temperature; each percentage shown is a percentage of that cell type over the total number of cells counted. The experiment was done in triplicate and the mean percentages are plotted; error bars indicate the standard deviation. Gray bars indicate *cdc4-3* cells; spotted bars, *cdc4-3 sic1*Δ cells. Times after the shift to the restrictive temperature are shown. (C) Flow cytometric analysis of cells at the indicated times after the shift to the restrictive temperature; histograms show DNA content on the horizontal axis and the number of counts on the vertical axis. (D) Micrographs of arrested *cdc4-3* and *cdc4-3 sic1*Δ cells showing bud morphology, DAPI-stained DNA, and Spc42-GFP-labeled SPBs. Scale bar: 2 µm.

To determine if Sic1 is the only SCF^Cdc4^ target important for SPB separation, we deleted *SIC1* in a strain carrying a temperature-sensitive *CDC4* allele, *cdc4-3* [19], [50], [51], and expressing GFP-tagged Spc42, a SPB component. We then asked if the ability of these mutant cells to separate SPBs at the restrictive temperature was restored. In agreement with earlier studies [20], [51], [52], we observed that an asynchronous culture of *cdc4-3* cells arrests almost uniformly at the G1/S border with elongated buds, unreplicated DNA, and duplicated but unseparated SPBs 2–4 hours after being shifted to 37°C (Figure 1). In contrast, an asynchronous culture of *cdc4-3 sic1*Δ arrests at 37°C with a majority of cells in G2/M with large, round buds and replicated DNA, as has been observed previously [20], [52]. Most importantly, for this study, duplicated and separated SPBs were observed in the majority (>90%) of *cdc4-3 sic1*Δ cells within 4 hours of the shift to the restrictive temperature (Figure 1). This finding indicates that the failure to separate SPBs in the absence of SCF^Cdc4^ activity is due solely to the stabilization of Sic1 and the consequent inhibition of Clb/Cdc28 kinase activity. These results are consistent with observations made by Goh & Surana who observed the formation of short spindles by immunofluorescence in asynchronous *GAL-CDC4 cdc4*Δ *sic1*Δ cells shifted to glucose to inhibit Cdc4 expression [53]. Those results were, however, inconclusive because the control cells (*P_GAL1_-CDC4 cdc4*) used in that particular study did not arrest at the G1/S border, potentially due to the persistence of a low level of Cdc4.

To address the possibility that the SCF^Cdc4^ may still play a role in bridge cleavage, meaning that duplicated SPBs might separate aberrantly in *cdc4-3 sic1* cells at the restrictive temperature, we examined the ability of *cdc4-3 sic1* cells to resume proliferation at permissive temperatures following prolonged arrest at 37°C. Should aberrant SPB separation occur in *cdc4-3 sic1* cells at the restrictive temperature, the separated SPBs may either lack half-bridges or have defective half-bridges, thus leading to subsequent delays in proliferation. We collected *cdc4-3 sic1* cells during the temperature shift experiment (described above) at various times after shifting to 37°C for spotting on solid media. Plates were subsequently incubated at permissive temperature (22°C). We found that although viability decreases as *cdc4-3 sic1* cells are maintained at 37°C over time, the colonies that do form show a similar range of sizes, regardless of exposure to the restrictive temperature (Figure S1A). There was also no evidence of SPB separation defects when the *cdc4-3 sic1* cells taken from these colonies were observed by fluorescence microscopy (Figure S1B).

### Kip1 and Cin8 are phosphorylated by Clb2/Cdc28 *in vitro*

**Figure 2:**
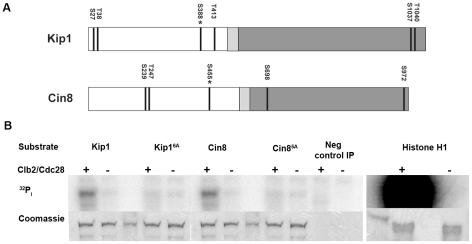
Phosphorylation of Kip1 and Cin8 by Clb2/Cdc28 *in vitro*. (A) Schematic showing the domain structure of Kip1 and Cin8 and the distribution of consensus CDK phosphorylation sites (S/T-P-X-X, indicated by dark lines) in each protein. The N-terminal motor domain is presented in white, the neck linker in light gray, and the C-terminal stalk and tail in dark gray; asterisk indicates the site that is conserved in almost all known kinesins-5 (Ser 388 in Kip1, Ser 455 in Cin8). (B) Wild-type Kip1 and Cin8, as well as their multiple consensus CDK site mutant forms (Kip1^6A^, Cin8^5A^) were immunoprecipitated from yeast lysates and mixed with soluble Clb2/Cdc28, also prepared from yeast, and ^32^P-γ-ATP. Soluble histone H1 (1.0 µg) was used as a control substrate. Proteins were subjected to SDS-PAGE after one hour at 30°C. PhosphorImages are shown on top and corresponding Coomassie-stained bands below. Unmarked lanes either contain molecular weight standards or had no protein loaded.

Based on existing genetic evidence in *S. cerevisiae*, and the finding that cyclin B/Cdk1 regulates centrosome separation in certain metazoan systems by phosphorylating the tail domain of kinesin-5 [35]–[40], we hypothesized that Clb/Cdc28 promotes SPB separation by regulating Kip1 and Cin8 function via direct phosphorylation. There are, however, six consensus CDK phosphorylation sites (S/T-P-X-X) in Kip1 and five in Cin8 (Figure 2A). Although a previous large-scale study by Ubersax et al. identified a large number of yeast proteins that are phosphorylated by Clb2/Cdc28, neither kinesin-5 was identified as a substrate [54]. The BimC box motif found in most kinesin-5 motors is phosphorylated in several metazoan systems but the motif is noticeably absent in both Kip1 and Cin8.

To determine if Kip1 and Cin8 can be phosphorylated by Clb/Cdc28 complexes, we carried out an *in vitro* phosphorylation assay. Kip1 and Cin8 tagged with 12 copies of the c-Myc epitope were expressed from the *GAL1* promoter in yeast cells expressing a hyperstabilized *SIC1* allele (*SIC1*Δ*3P*) [49] to inhibit phosphorylation by Clb/Cdc28. We also expressed the mutants Kip1^6A^ and Cin8^5A^, in which the serine or threonine of every consensus CDK site is mutated to non-phosphorylatable alanine. Wild-type and mutant kinesins were immunoprecipitated separately with anti-c-Myc IgG-agarose beads and then mixed with soluble Clb2/Cdc28 kinase and ^32^P-γ-ATP.

Both Kip1-myc_12_ and Cin8-myc_12_ were phosphorylated in a reproducible manner by Clb2/Cdc28 *in vitro* (Figure 2B). Furthermore, Kip1^6A^-myc_12_ was, on average, almost 2–3 fold less phosphorylated compared with wild-type Kip1, whereas Cin8^5A^-myc12 was not phosphorylated above background levels. This observation indicates that phosphorylation of Kip1 by Clb/Cdc28 can occur at sites other than the six consensus CDK sites. We also determined that the observed phosphorylation is likely to be specific to mitotic CDK complexes as a similarly prepared S-phase CDK complex, Clb5/Cdc28, did not phosphorylate either Kip1-myc_12_ or Cin8-myc_12_ to any significant extent under similar reaction conditions (Figure S2).

### Clb/Cdc28 kinase activity does not regulate Kip1 and Cin8 protein abundance

Both Kip1 and Cin8 are thought to be targeted to the proteasome by APC (anaphase promoting complex)-mediated ubiquitination [46], [47]. It has been proposed that Clb/Cdc28 controls SPB separation indirectly by regulating the *in vivo* stability of the Kip1 and Cin8 proteins [42], [55]. These findings were based on strains carrying either of two mutant *CDC28* alleles. The first allele bears a mutation of tyrosine 19 to glutamate that mimics an inhibitory phosphorylation (*cdc28^Y19E^*) [10], [11] and the second is a conditional mutant (*cdc28-as1*) which is inhibited by the ATP analog, 1-NM-PP1 [48]. However, both *cdc28^Y19E^* strains, and *cdc28-as1* strains (at the concentration of analog used in these studies) are still capable of DNA replication [12], [41], [48], indicating that these alleles still retain some CDK activity.

**Figure 3:**
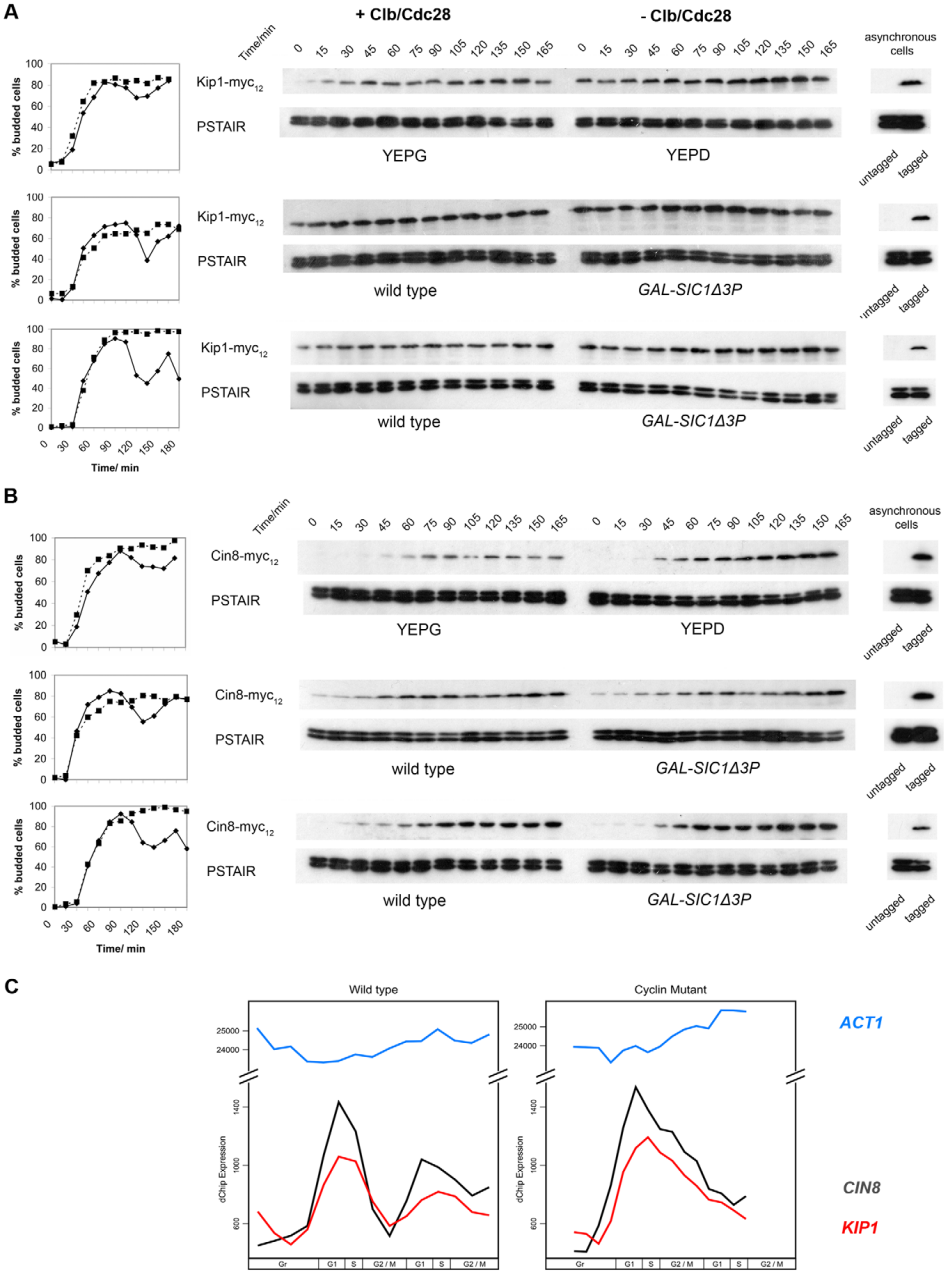
Kip1 and Cin8 protein and mRNA levels in the presence and the absence of active Clb/Cdc28 kinase. Kip1 and Cin8 protein levels were determined by immunoblotting (see text) in three different sets of strains that were first synchronized with α-factor: BF264-15DU *P_GAL1_-CLB1* Δ*clb1*, *2*, *3*, *4*, *5*, *6* cells released into YEPG versus YEPD (top panels); wild-type BF264-15DU cells and mutants overexpressing Sic1Δ3P (*GAL-SIC1*Δ*3P*) to specifically inhibit Clb/Cdc28 (middle panels); wild-type W303a cells and mutants overexpressing Sic1Δ3P (bottom panels). Budding kinetics and corresponding immunoblots for strains with c-Myc-tagged Kip1 (A) and Cin8 (B) are shown; anti-PSTAIR was used as a loading control. Key: budding data for strains with active Clb/Cdc28 kinase (♦); lacking Clb/Cdc28 kinase activity (▪). (C) Transcript levels for *KIP1* (red), *CIN8* (black), and *ACT1* (light blue) determined in wild-type and Δ*clb1-6* BF264-15DU cells synchronized in early G1 using centrifugal elutriation as reported in Orlando et al. (2008) [56].

Hence, we determined the levels of Kip1 and Cin8 protein in strains deleted for all the B-type cyclin genes, as well as in a separate set of strains that overexpress the hyperstabilized *SIC1* allele, *SIC1*Δ*3P* [49]. Phenotypic data indicate that these strains lack all Clb/Cdc28 activity as the cells are unable to either initiate DNA replication or enter mitosis [4], [20], [49], [56]. Cells were arrested in G1 with α-factor, and subsequently released into the appropriate medium to eliminate Clb/Cdc28 kinase activity. We observed that Kip1 (Figure 3A) and Cin8 (Figure 3B) protein levels were stable over time in the absence of Clb/Cdc28 kinase activity. In order to verify that our findings are not strain-specific, we repeated our *GAL-SIC1*Δ*3P* experiment in a *W303a* strain background. Just as we had observed in our own strain background, Kip1 and Cin8 protein levels did not change significantly in *W303a* cells lacking Clb/Cdc28 kinase activity (Figure 3). We verified that BF264-15DU and W303a *GAL-SIC1*Δ*3P* strains both arrested with unreplicated DNA and unseparated SPBs (Figure S3). Furthermore, persistent levels of Kip1 and Cin8 do not reflect de-regulated transcription, as *KIP1* and *CIN8* mRNAs accumulate periodically in Δ*clb1,2,3,4,5,6* mutant cells [56] (Figure 3C).

### Clb/Cdc28 kinase activity does not regulate the localization of Kip1 and Cin8 to the spindle

In certain metazoan systems, the phosphorylation of a consensus CDK site located on the tail domain is required for the localization of kinesin-5 motors to the spindle [35]–[40]. Although this site is absent from the tails of both *S. cerevisiae* kinesins-5, regions of the Kip1 and Cin8 tail domains have been found to be important to their localization to the nucleus [46], [47], [57]. Moreover, there are two consensus CDK sites (Ser 1037 and Thr 1040) found within the smallest defined nuclear localization sequence (NLS) on the Kip1 tail [46], and one (Ser 972) just N-terminal to the reported Cin8 NLS [47]. Thus, it is possible that Kip1 and Cin8 localization is regulated by Clb/Cdc28-mediated phosphorylation of these residues.

**Figure 4:**
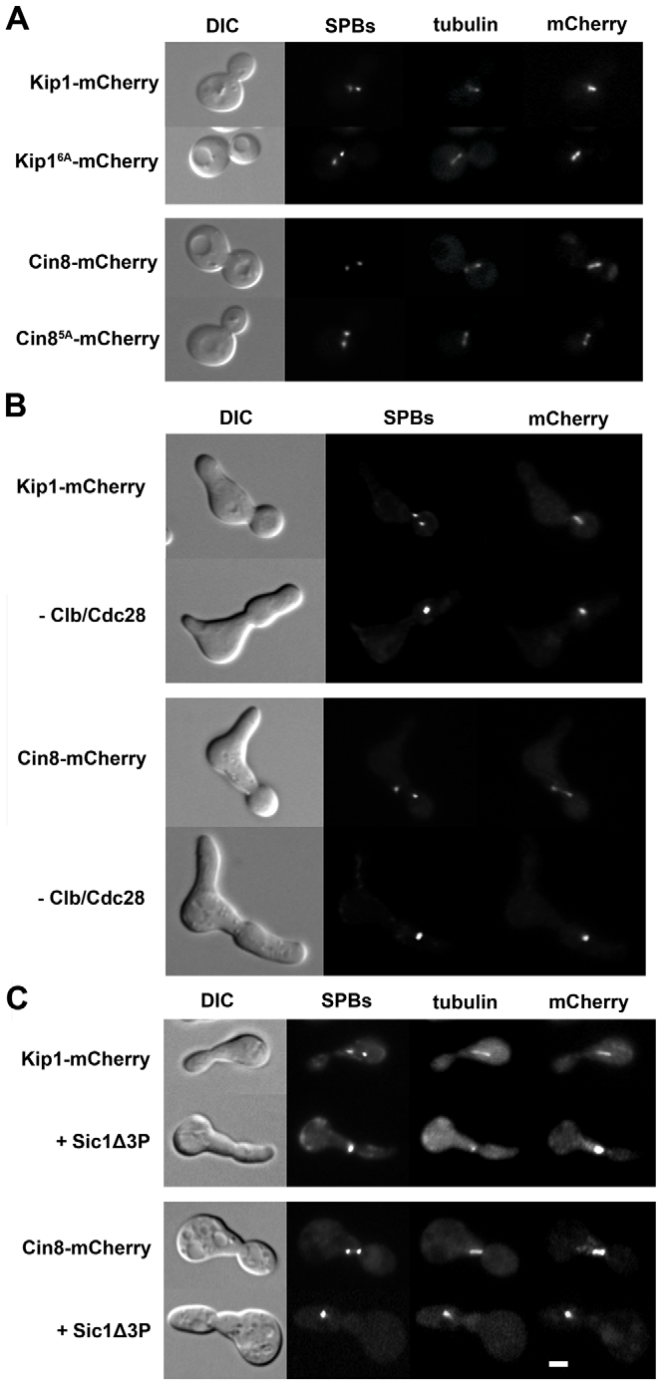
Kip1 and Cin8 localization in the presence and the absence of phosphorylation by Clb/Cdc28. Kip1 and Cin8 were visualized by fusion to a C-terminal mCherry tag and imaging with fluorescence microscopy. SPBs in the strains shown are marked with Spc42-GFP, and microtubules with CFP-Tub1. (A) Localization of wild-type Kip1 (top) and Cin8 (bottom), compared with that of Kip1^6A^ and Cin8^5A^ mutants, in live yeast cells. All consensus CDK sites in the mutant kinesins-5 have been mutated to non-phosphorylatable alanine. (B) Kip1 and Cin8 localization in *P_GAL1_-CLB1* Δ*clb1*, *2*, *3*, *4*, *5*, *6* cells 90 minutes after being released from α-factor arrest into medium containing either galactose (top panel; to induce Clb1 expression) or dextrose (bottom panel; to inhibit Clb1 expression). (C) Kip1 and Cin8 localization in wild-type cells versus cells with *P_GAL1_*-*SIC1*Δ*3P* integrated, 90 minutes after being released from α-factor arrest into galactose medium. Scale bar: 2 µm.

**Figure 5:**
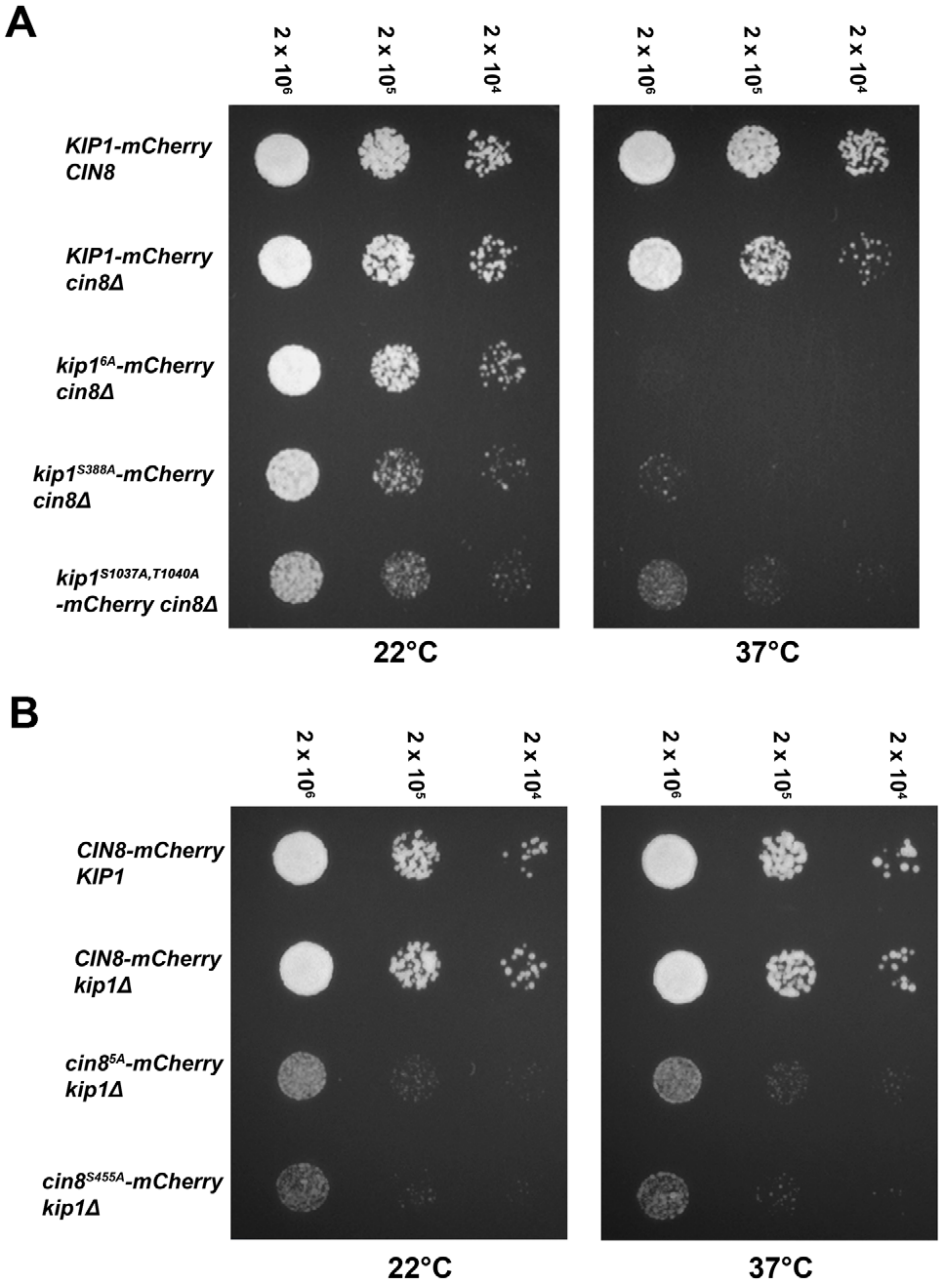
Impaired proliferation of strains with CDK site point mutants (Ser/Thr→Ala) of either (A) Kip1 or (B) Cin8 as their only kinesin-5. Strains growing in log phase at permissive temperature were diluted to 2×10^6^ cells/ml, and then further diluted serially to 2×10^4^ cells/ml. An equal volume of cells from each dilution was spotted on YEPD, and plates were incubated at either ambient temperature (∼22°C) or 37°C. The number above each column of spots indicates the cell density (cells/ml). All alleles compared were tagged with mCherry at their C-terminus.

To address this possibility, C-terminal mCherry [58] tags were fused to Kip1 and Cin8 expressed from their respective native promoters, and their localization was determined by fluorescence microscopy (Figure 4). We determined that Kip1-mCherry and Cin8-mCherry are both functional as they both supported growth in a *kip1*Δ *cin8*Δ background (Figure 5). We observed that wild-type Kip1 and Cin8, as well as the non-phosphorylatable mutants, Kip1^6A^ and Cin8^5A^, localized to the spindle poles and spindle microtubules (Fig 4A). These findings suggest that phosphorylation of Kip1 and Cin8 at their consensus CDK sites is not required for spindle localization.

Clb/Cdc28 has been shown, however, to phosphorylate serine and threonine residues that do not match the consensus Ser/Thr-Pro motif [49], [59], [60]. In order to determine if Clb/Cdc28 kinase activity is necessary for the spindle localization of Kip1 and Cin8, we examined localization both in Δ*clb1,2,3,4,5,6* and in separate *GAL1-SIC1*Δ*3P* strains. Cells were arrested in G1 with α-factor, and then released into medium containing either dextrose (Δ*clb1,2,3,4,5,6* strains) or galactose (*P_GAL1_-SIC1*Δ*3P* strains) in order to eliminate Clb/Cdc28 kinase activity. The *KIP1* and *CIN8* genes were fused at their native loci to mCherry and localization of the tagged proteins was monitored by fluorescence microscopy (Figure 4B and 4C). Both Kip1-mCherry and Cin8-mCherry still localized to the SPBs, even in the absence of active Clb/Cdc28 kinase. Taken together, these findings suggest that Clb/Cdc28 complexes do not control SPB separation by regulating the localization of Kip1 and Cin8 to the spindle.

### Mutation of consensus CDK sites in Kip1 and Cin8 impairs cell proliferation

To determine if Clb/Cdc28 phosphorylation of Kip1 and Cin8 may regulate other motor functions essential to cell division, we examined the proliferation of cells bearing the non-phosphorylatable *kip1^6A^* and *cin8^5A^* alleles using a spot assay (Figure 5). As Kip1 and Cin8 are partially redundant [24], [25], we examined the motor mutants in a strain background where the other motor was deleted. Proliferation was assayed at both ambient temperature (∼22°C) and at 37°C as strains deleted for the *CIN8* gene have been reported to exhibit temperature-sensitive growth [24], [25]. We observed that although *kip1^6A^-mCherry cin8*Δ cells proliferated at a rate similar to that of *KIP1-mCherry cin8*Δ cells at ambient temperature, they failed to form colonies at 37°C (Figure 5A). In contrast, although *cin8^5A^-mCherry kip1*Δ cells also proliferated more slowly compared to *CIN8-mCherry kip1*Δ cells, this defect was not a temperature-sensitive one (Figure 5B). We verified that the observed defects were not caused by the mCherry tag by examining strains expressing untagged Kip1^6A^ and Cin8^5A^ as their sole kinesin-5 (Figure S4).

To determine which of the CDK consensus site mutations contributed to the proliferation defect, we assayed the proliferation of cells containing individual mutations in each of the six consensus CDK phosphorylation sites in Kip1. The substitution of Ser 388 with alanine alone was sufficient to cause temperature-sensitive lethality in the absence of Cin8 (Figure 5A); no other single CDK site when similarly mutated caused temperature-sensitive lethality (data not shown).

Mutating the homologous serine residue in Cin8, Ser 455, to alanine was sufficient to cause the proliferation defect associated with Cin8^5A^ in the absence of Kip1 (Figure 5B). Similarly to Kip1, mutating any other single CDK site on Cin8 to alanine did not cause the Cin8^5A^ growth defect. As a control, we also verified that the mCherry tag was not responsible for causing the proliferation defect associated with either Kip1^S388A^ or Cin8^S455A^ (Figure S4).

Although we were unable to test all the effects of mutating all possible combinations of the individual consensus CDK sites on Kip1 and Cin8, we did test tandem mutations of CDK sites located in close proximity to each other in the primary structure of the two proteins. In doing so, we found that, *kip1^S1037A, T1040A^ cin8*Δ cells proliferated at a significantly slower rate than cells bearing a wild-type copy of *KIP1* (Figure 5A and S4A). Unlike *kip1^S388A^ cin8*Δ cells, however, *kip1^S1037A, T1040A^ cin8*Δ cells were not fully arrested at 37°C.

### The role of kinesin-5 phosphorylation in SPB separation

**Figure 6:**
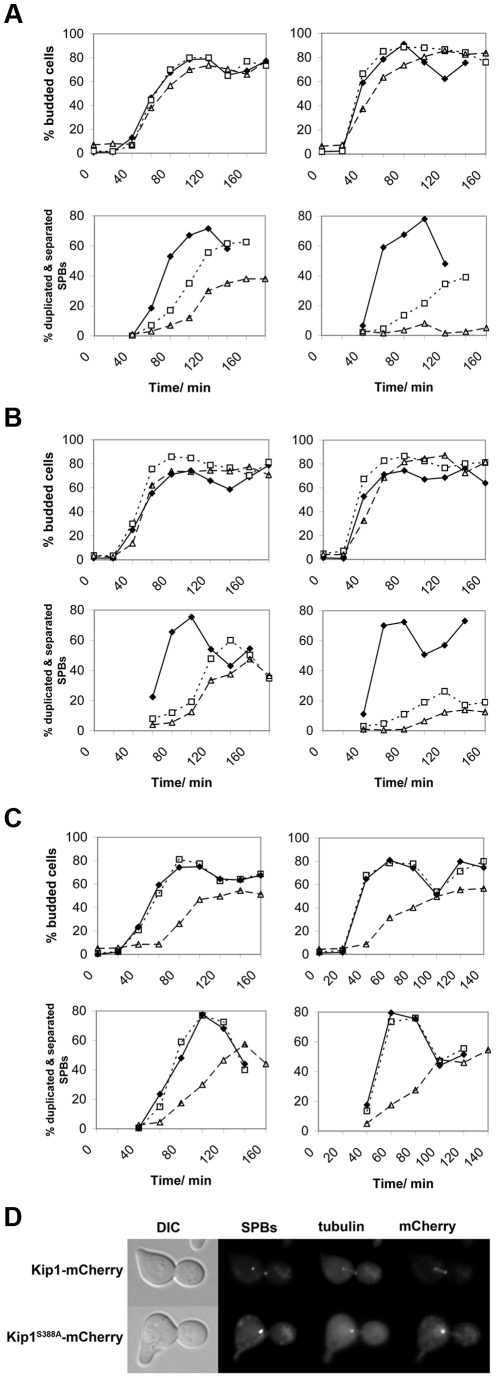
Impaired SPB separation in cells dependent on either Kip1 or Cin8 CDK site mutants as their only kinesin-5. Cells were first synchronized in G1 with α-factor, and then released at either 25°C (permissive temperature, left column) or 37°C (restrictive temperature, right column). Time course experiments were carried out in triplicate, and both the percentage of budded cells (top panels for each group of strains) and the percentage of cells with duplicated and separated SPBs (bottom panels) from representative experiments are shown. The time elapsed following release from α-factor is indicated in minutes on the horizontal axis of each graph. (A) Cells expressing wild-type Kip1 and Cin8 (♦), cells expressing only Kip1 (□, dotted line) cells, and cells expressing only Kip1^S388A^ (▵, dashed line); (B) cells expressing wild-type Kip1 and Cin8 (♦), cells expressing only Kip1 (□, dotted line) cells, and cells expressing only Kip1^S1037A, T1040A^ (▵, dashed line); (C) cells expressing wild-type Cin8 and Kip1 (♦), cells expressing only Cin8 (□, dotted line), and cells expressing only Cin8^S455A^ (▵, dashed line). (D) Fluorescence images of representative *KIP1 cin8*Δ and *kip1^S388A^ cin8*Δ cells at 37°C 100 min after being released from α-factor arrest during the timecourse described in (A). Scale bar: 2 µm.

To determine if the proliferation defects associated with the *kip1^S388A^* and *kip1^S1037A, T1040A^* alleles are related to a defect in SPB separation, we arrested cells in G1 with α-factor at 25°C. Cells were subsequently released at either 25°C or 37°C (restrictive temperature) and SPB separation was monitored over time by fluorescence microscopy. While *kip1^S388A^ cin8*Δ cells budded at a rate similar to *KIP1 cin8*Δ cells at both temperatures tested, most *kip1^S388A^ cin8*Δ cells were unable to separate SPBs at 37°C (Figure 6A). Even more than two hours after being released from α-factor arrest, less than 10% of *kip1^S388A^ cin8*Δ cells had separated their SPBs at 37°C, compared to more than 40% of *KIP1 cin8*Δ cells. A clear SPB separation defect was also observed for *kip1^S1037A, T1040A^ cin8*Δ cells at 37°C (Figure 6B). At 25°C, *cin8*Δ cells expressing Kip1^S388A^ appear to be impaired to a greater extent in SPB separation than cells expressing Kip1^S1037A, T1040A^ compared with *cin8*Δ cells expressing wild-type Kip1, which is consistent with the more severe proliferation defect observed for *kip1^S388A^ cin8*Δ cells (Figure 5A and Figure S4A).

Although unlikely [61], the single S388A and tandem S1037A, T1040A mutations may cause the loss of Kip1 function by perturbing the protein's *in vivo* stability. To address this possibility, we compared the levels of Kip1^S388A^-mCherry and Kip1^S1037A, T1040A^-mCherry in separate time courses with that of Kip1-mCherry using cells synchronized in α-factor and released at 37°C by western blotting (Figure S5A). In doing so, we verified that both mutant and wild-type protein levels were similar at the restrictive temperature. In addition, as expected from the observed localization of Kip1^6A^, both Kip1^S388A^ and Kip1^S1037A, T0140A^ continued to localize to the SPBs and spindle microtubules even at 37°C (Figure S5B). Taken together, our findings suggest that the phosphorylation of Ser 388 by Clb/Cdc28 is critical for Kip1-mediated separation of SPBs, while the phosphorylation of Ser 1037 and Thr 1040 also contributes to the function of Kip1.

Similar analyses of non-phosphorylatable *cin8* alleles were performed, although we were unable to determine conclusively if there is a SPB separation defect in *cin8^5A^ kip1*Δ and *cin8^S455A^ kip1*Δ cells. Although *cin8^S455A^ kip1*Δ cells showed a delay in SPB separation (Figure 6C) after release from α-factor, they also appeared to have an uncharacterized defect in progression through G1 as the initiation of both bud emergence (Figure 6C) and DNA replication (data not shown) were both significantly delayed after release from α-factor. Hence, we cannot confirm that Cin8 phosphorylation at Ser 455 plays a role in controlling SPB separation due to the potential confounding effects of the apparent G1/S phase delay.

As expected, flow cytometric analyses of DNA content demonstrated that at 37°C, asynchronous populations of *cin8*Δ cells expressing either Kip1^6A^ or Kip1^S388A^ were enriched for cells with replicated DNA (Figure S6A), supporting the observation that these cells have a defect in mitotic spindle assembly. However, asynchronous log phase populations of *kip1*Δ cells expressing either Cin8^5A^ or Cin8^S455A^ were clearly enriched for cells with unreplicated DNA compared to *kip1*Δ cells expressing wild-type Cin8 at 37°C (Figure S6B). These analyses confirm that putative CDK phosphorylation site mutations in Cin8 give rise to a defect in the G1/S phase transition. The nature of this defect is unknown.

### Kip1 and Cin8 are phosphorylated *in vivo* in a Clb/Cdc28-dependent manner

Given the genetic and *in vitro* biochemical evidence we had garnered, we sought to determine if Kip1 and Cin8 are indeed phosphorylated by Clb/Cdc28 complexes *in vivo* using mass spectrometry. Kip1-myc_12_ and Cin8-myc_12_ expressed from the *GAL1* promoter in yeast cells for subsequent immunopurification. Additionally, we also expressed the two kinesins-5 in yeast that also expressed either Sic1Δ3P to inhibit Clb/Cdc28 kinases or Clb2-HA_3_ to promote phosphorylation of Clb/Cdc28 substrates. Immunopurified Kip1-myc_12_ and Cin8-myc_12_ were subject to SDS-PAGE followed by protein phosphorylation analysis using microcapillary LC/MS/MS techniques.

Kip1 and Cin8 were determined to both be phosphorylated *in vivo* at multiple residues in all the samples. A total of eight phosphorylation sites were assigned with high confidence for Kip1 and four were assigned for Cin8 (Table 1; Sequest Xcorr values are available upon request). Although not all of the phosphorylated residues were observed in all the samples, many of the phosphopeptides generated had phosphate(s) assigned to the same residue. Of particular interest to us were the phosphorylations assigned to consensus CDK sites located in the tail domains of the two kinesin-5 motors, namely residues Ser 1037 and Thr 1040 in Kip1, and Ser 972 in Cin8. For Kip1, these sites are particularly relevant since we observed proliferation and SPB separation defects when both Ser 1037 and Thr 1040 were mutated in tandem to Ala (Figure 5 and 6).

**Table 1:** List of phosphopeptides generated from Kip1 and Cin8 and identified during LC/MS/MS analysis.

Sequence of Identified Peptide	Phosphorylation site(s) assigned	Co-overexpression		
		+*Sic1*Δ*3P*	None (wild-type)	+Clb2
**Kip1**				
ISFLELYNENLKDLLS*DSEDDDPAVNDPK	Ser 213	**−**	**−**	**+**
SFLELYNENLKDLLS*DSEDDDPAVNDPK	Ser 213	**+**	**−**	**−**
DLLS*DSEDDDPAVNDPK	Ser 213	**+**	**+**	**−**
IHSDS*IASLAHNAENTLK	Ser 802	**−**	**+**	**−**
TQDEVLS*EHCEK	Ser 952	**−**	**+**	**−**
TCIPNLSTNENFPLSQFS*PK	***Ser 1037***	**+**	**+**	**+**
T*PVPVPDQPLPK	***Thr 1040***	**−**	**+**	**−**
TCIPNLSTNENFPLSQFS*PKTPVPVPDQPLPK	***Ser 1037***	**−**	**−**	**+**
TCIPNLSTNENFPLSQFSPKT*PVPVPDQPLPK	***Thr 1040***	**+**	**−**	**−**
TCIPNLSTNENFPLSQFS*PKT*PVPVPDQPLPK	***Ser 1037***, ***T1040***	**+**	**+**	**+**
SINS*AK	Ser 1060	**+**	**−**	**+**
SKT*LPNTEGTGR	Thr 1068	**+**	**+**	**−**
RFT*TEPILK	Thr 1088	**+**	**+**	**+**
**Cin8**				
IFDSSTANNTTSNS*ASSSR	Ser 227	**+**	**−**	**−**
TKS*LPNTIK	Ser 261	**+**	**−**	**+**
KS*LPNTIK	Ser 261	**−**	**+**	**−**
S*LPNTIK	Ser 261	**−**	**+**	**−**
LSNINSNSVQSVIS*PK	***Ser 972***	**+**	**+**	**+**
KHAIEDENKS*SENVDNEGSR	Ser 984	**+**	**+**	**−**

Kip1-myc_12_ and Cin8-myc_12_ were overexpressed either alone in yeast cells (wild-type), co-overexpressed with Sic1Δ3P to inhibit Clb/Cdc28 complexes (+Sic1Δ3P) or co-overexpressed with Clb2 (+Clb2). The assigned phosphoresidues are shown in bold and indicated with an asterisk (*); CDK sites are indicated with bold italics. A plus (+) indicates that the phosphopeptide was observed while a minus (−) indicates that it was not observed.

**Figure 7:**
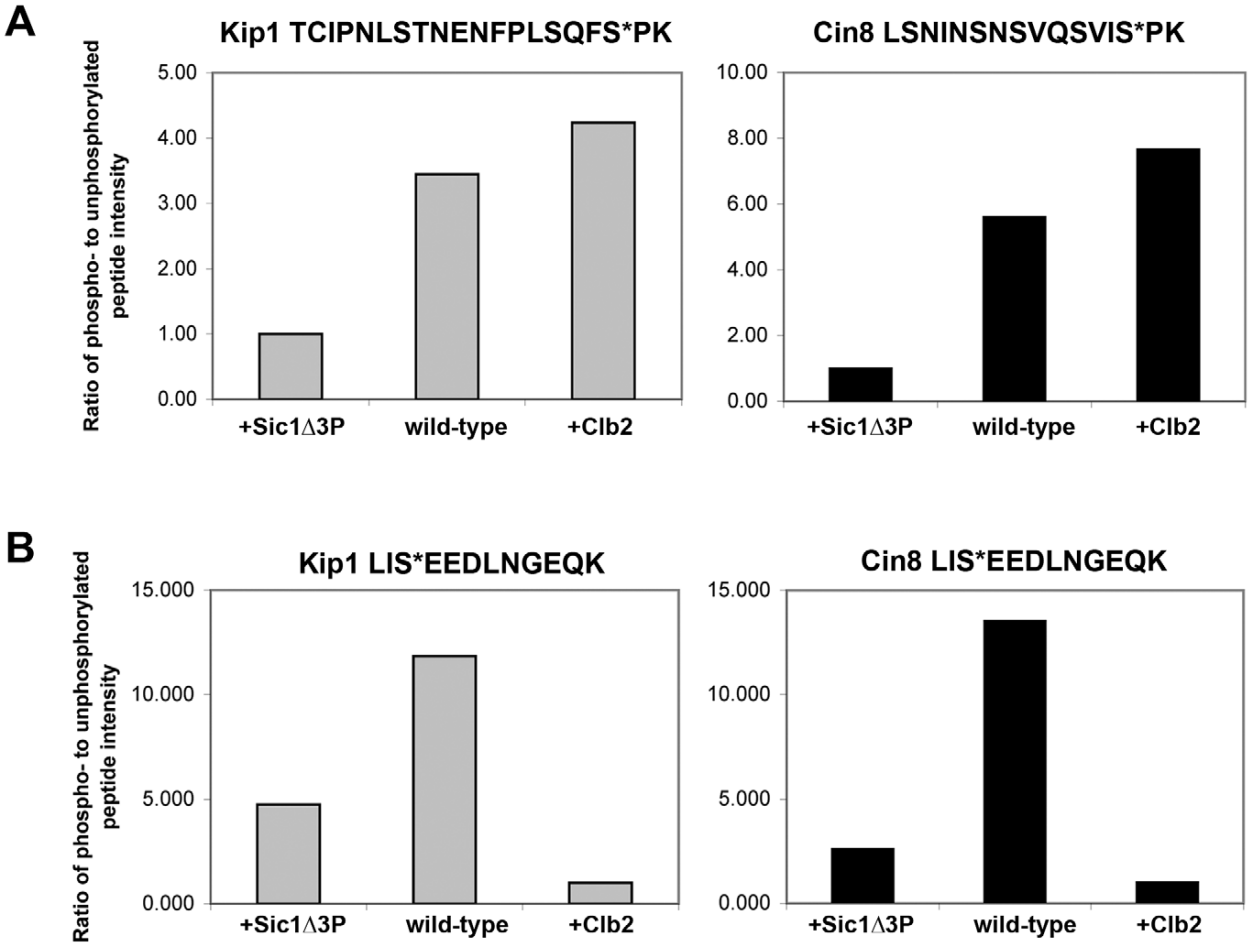
Relative abundance of specific phosphopeptides generated from Kip1-myc_12_ and Cin8-myc_12_ purified from yeast as measured by LC/MS/MS. (A) Ratio of the intensity levels of the peaks corresponding to phosphopeptides with phosphates assigned to consensus CDK phosphorylation sites to that of their respective unphosphorylated forms. TCIPNLSTNENFPLSQFS*PK (asterisk denotes Ser 1037) was derived from Kip1 (gray bars), and LSNINSNSVQSVIS*PK (asterisk denotes Ser 972) from Cin8 (black bars). Kip1-myc_12_ and Cin8-myc_12_ were expressed from the *GAL1* promoter in yeast (wild-type) for mass spectrometry analysis, and were also expressed in strains that simultaneously expressed either Sic1Δ3P (+Sic1Δ3P) or Clb2-HA_3_ (+Clb2) from the *GAL1* promoter. (B) Relative intensity levels of peaks corresponding to phosphorylated and unphosphorylated forms of the peptide LIS*EEDLNGEQK, derived from the c-Myc epitope tags of Kip1-myc_12_ (gray bars) and Cin8-myc_12_ (black bars).

In addition, the LC/MS/MS analysis yielded peak intensities (Table S1) which enabled us to compare the relative abundance of phosphopeptides common to all three samples submitted for each protein with that of their unphosphorylated forms. In doing so, we determined that the extent of phosphorylation of TCIPNLSTNENFPLSQFSPK (containing Ser 1037, underlined) from Kip1 and LSNINSNSVQSVISPK (containing Ser 972, underlined) from Cin8 were both greatly reduced in the presence of overexpressed Sic1Δ3P but noticeably increased in the presence of overexpressed Clb2 (Figure 7A), lending support to our hypothesis that Kip1 and Cin8 are both phosphorylated by Clb/Cdc28. As a control, we examined the relative phosphorylation of a non-CDK substrate, the c-Myc epitope tag of both Kip1-myc_12_ and Cin8-myc_12_ which was determined to be phosphorylated on the serine residue of each LISEED motif. We verified that the phosphorylation of the c-Myc tag was not CDK-dependent as the c-Myc epitope was the least phosphorylated in cells overexpressing Clb2 (Figure 7B).

Although we were unable to identify phosphopeptides containing Ser 388 from Kip1 or Ser 455 from Cin8, it is often difficult to capture the full extent of protein phosphorylation during mass spectrometry analysis due to both technical and biological limitations [62], [63]. In addition to the phosphopeptides which contain consensus CDK sites, other phosphopeptides were generated from Kip1 and Cin8 that did not include consensus CDK phosphorylation motifs (Table 1; kinases predicted to phosphorylate these non-CDK sites are listed in Table S1 and details of the prediction method are in Text S1). Thus, the results of our mass spectrometry analysis do not rule out the possibility that Kip1 and Cin8 are also phosphorylated at other sites by Clb/Cdc28 complexes.

### Homology modeling of Kip1 and Cin8 motor domains

**Figure 8:**
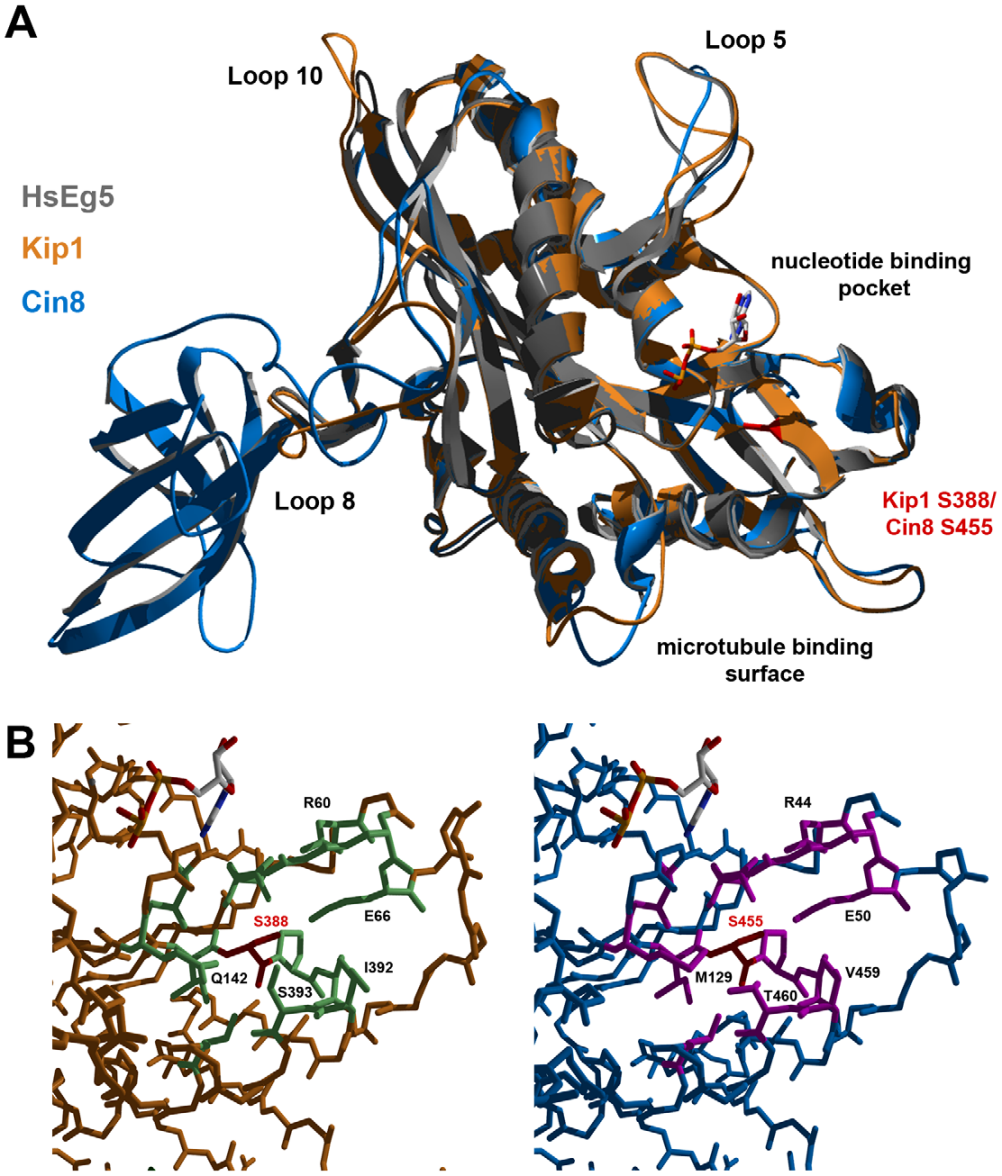
Homology models of Kip1 and Cin8 motor domains bound to MgADP. (A) Ribbon structure overlay illustrating gross differences in the predicted structures of Kip1 (peach) and Cin8 (light blue), particularly in the length of the various loop regions. The human Eg5 (also known as Kif11) template structure (ExPDB 1ii6B) is shown in gray. The position of Ser 388 on Kip1 and Ser 455 on Cin8 is highlighted in red, while the location of the microtubule binding face and nucleotide binding pocket are indicated. (B) Close-up view of the surroundings of Ser 388 in Kip1 and Ser 455 in Cin8; both the serine residues are colored red. Residues with side chains lying within a 2.5 Å radius of the serine residue are colored light green for Kip1 and hot pink for Cin8; residues have also been labeled to highlight significant differences between the two model structures.

Ser 388 in Kip1 and Ser 455 in Cin8 are found in the N-terminal motor domain of the respective kinesins-5. Hence, in order to understand how phosphorylation at these residues on Kip1 and Cin8 might regulate their functions, we constructed homology models of their motor domains (Figure 8A). Homology modeling was performed using SWISS-MODEL and Swiss PDB Viewer [64]–[66] with X-ray crystal structures of the motor domains of human kinesin-5 HsEg5 [67] and the budding yeast kinesin-14 Kar3 [68] serving as templates. Consistent with the idea that phosphorylation of Ser 388 could regulate motor function, our model of the Kip1 motor domain showed that Ser 388 is solvent-accessible and located at the C-terminal end of strand β8. Here, Ser 388 appears to form part of the core which enables the motor to distinguish between ATP and ADP bound to the nucleotide-binding pocket [69]. The residue itself, however, is not predicted to form essential hydrogen bonds, and does not itself form part of the nucleotide binding pocket. Additional modeling (see Materials & Methods) showed that replacing Ser 388 with an alanine residue has no predicted effects on the backbone structure.

The Cin8 motor domain was modeled on the same template structures as for Kip1. Ser 455 in Cin8 is found in a similar environment to Ser 388 in Kip1, as expected. Closer scrutiny of its neighboring amino acids, however, revealed critical differences (Figure 8B). These differences include the residue at the second position of the P-loop, which is glutamine in Kip1 and Eg5 but methionine in Cin8, and also the two residues on helix α6 (C-terminal to strand β8) closest to Ser 455 (Val 459, Thr 460) and Ser 388 (Ile 392, Ser 393).

## Discussion

Previous genetic studies indicate that kinesin-5 motors, Clb/Cdc28 complexes, and the SCF complex are all required for SPB separation and assembly of a mitotic spindle. We have shown that the only important target for SCF^Cdc4^ in SPB separation is the Clb-specific CDK inhibitor, Sic1 (Figure 1). This finding indicates that SCF-mediated destruction of other regulatory proteins or SPB components is not required for SPB separation, and also supports the idea that Clb/Cdc28 complexes promote SPB separation and spindle assembly via regulation of kinesin-5 motors. We have, in fact, determined that kinesin-5 motors are phosphorylated directly by Clb/Cdc28 complexes (Figure 2 and Table 1) and that this phosphorylation plays a role in promoting SPB separation and spindle assembly (Figure 6).

Although certain metazoan kinesin-5 orthologues have been shown to be phosphorylated on their tail domains by cyclin B/Cdk1 [35], [37], [39], the threonine residue at which this phosphorylation occurs is absent from both Kip1 and Cin8. We have shown, however, that Kip1 and Cin8 are both phosphorylated by Clb2/Cdc28 *in vitro*, and that mutation of their consensus CDK sites significantly reduces the extent of phosphorylation (Figure 2). By genetic mapping, we have identified a solvent-accessible consensus CDK site (Ser 388) in the motor domain of Kip1 that is crucial to its role in SPB separation. Additionally, using a combination of mass spectrometry and genetic analysis, we have found that the phosphorylation of two consensus CDK sites in the tail domain of Kip1 is also important for timely SPB separation. Cells dependent on Kip1^S388A^ or Kip1^S1037A, T1040A^ as their only source of kinesin-5 are severely impaired in their ability to separate duplicated SPBs (Figure 6). Both Kip1 mutants have similar *in vivo* protein levels to wild-type Kip1 at 37°C and still localize to the SPBs in arrested cells (Figure S5). Hence, we propose that Clb/Cdc28-mediated phosphorylation of Ser 388, Ser 1037, and Thr 1040, regulates some aspect of Kip1 motor activity.

Cells with the homologous serine in Cin8 (Ser 455) substituted with alanine exhibit a severe proliferation defect in the absence of Kip1 which appears to reflect a delay in the transition from G1 into S phase (Figure 6 and S6). Although we do observe an SPB separation defect in *cin8^S455A^ kip1*Δ mutants, the observed G1/S delay confounds our ability to determine if the separation defect is directly related to the loss of Cin8 motor function. The mechanism underlying this delay has yet to be determined, and the basis for the phenotypic differences between the homologous mutations in Kip1 and Cin8 remains unclear; however, homology modeling suggests that there are important structural differences between the two motors in the vicinity of this consensus phosphorylation site that might contribute to the different phenotypes observed.

Ser 388/Ser 455 forms part of the core of the motor domain, which includes the nucleotide-sensing elements switch I and switch II, and the γ-phosphate-sensing P-loop [69]. Due to the fact that Ser 388/Ser 455 lies near the junction of strand β8, helix α6, and the neck linker (Figure 8), this residue appears to be in a position to influence the transmission of structural changes in the motor core to the neck should it be phosphorylated. We have also noticed that although switch I and switch II are identical in Kip1 and Cin8, Cin8 has a methionine residue (Met 129) in the 2nd position of its P-loop whereas Kip1, like almost all other known kinesins-5, has a glutamine (Gln 142) at this position. The residues on α6 closest to Ser 455 in Cin8 (Val 459, Thr 460) also differ from those closest to Ser 388 in Kip1 (Ile 392, Ser 393). All of these differences are potentially important because these residues are close enough in space to interact; additionally, communication between the respective secondary structural elements to which they belong is essential to the generation of motility.

The Cin8 motor domain has a number of other notable structural differences compared with Kip1 and other kinesins-5. Loop L2 and, in particular, loop L8, are substantially longer in Cin8, while other loops such as L5 and L10 are shorter; the functional significance of these differences in length are still unclear. We do know, however, that L2 and L8 form part of the microtubule-binding surface of kinesins [70], [71] while the structure of L5 is thought to be important in determining ADP-release kinetics during the ATP hydrolysis cycle of Eg5 [72]. In addition to these differences in the structure of their motor domains, *CIN8*, but not *KIP1*, has been reported to be involved in a myriad of genetic and physical interactions [73]–[78], suggesting that Cin8 has several cellular functions. The dissection of these multiple functions in future studies may reveal a mechanistic role for direct phosphorylation in spindle assembly.

Additionally, although we were able to determine that Cin8 is phosphorylated at a CDK site in its tail domain, we have yet to observe a phenotype for *cin8^S972A^ kip1*Δ cells. Previous studies have also been unable to determine a phenotypic consequence for the same mutation [47], [57]. The distinct structure of the tails of Kip1 and Cin8 and their different responses to Clb/Cdc28 phosphorylation may also help explain the existence of two kinesins-5 in *S. cerevisiae* when many other eukaryotes appear to only have one.

It has been proposed that Clb/Cdc28 kinase activity regulates SPB separation indirectly by inhibiting the activity of the ubiquitin ligase, APC^Cdh1^, thus preventing the ubiquitination of Kip1 and Cin8 and their subsequent degradation [41], [42]. The proposal for Clb/Cdc28 regulation of Kip1 and Cin8 stability was based, however, on observations made with strains bearing the *cdc28^Y19E^* allele, and with strains carrying the analog-sensitive *cdc28-as1* allele treated with 500 nM 1-NM-PP1. In both cases, a sufficient level of Clb/Cdc28 activity remains to drive DNA replication [12], [41], [48]. By using more stringent means of inhibiting Clb/Cdc28 complexes, we have not observed evidence suggesting that the loss of Clb/Cdc28 activity leads to a decreased abundance of Kip1 and Cin8. Inhibiting Clb/Cdc28 activity by overexpressing a stabilized allele of Sic1 [49] in a W303 strain background similar to that used by Crasta et al. [41], [42] did not affect the abundance of either Kip1 or Cin8. Our observations suggest that the alterations in motor stability observed by Crasta et al. may reflect partially deregulated CDK activity rather than a loss of Clb/Cdc28 activity.

A new study by Robbins & Cross [79] found that most cells whose endogenous *CDH1* gene has been replaced with a non-Cdk1-phosphorylatable allele, *CDH1-m11* arrest with monopolar spindles, and that Cin8 levels are reduced about fourfold compared with *CDH1* cells. However, expressing non-degradable Cin8 at endogenous levels in *CDH1-m11* cells failed to drive SPB separation and bipolar spindle assembly. Instead, by expressing non-degradable Clb2, which is also an APC^Cdh1^ target, the authors observed the restoration of SPB separation in *CDH1-m11* cells. This observation led Robbins & Cross to conclude that the mitotic cyclins alone are the APC^Cdh1^ targets important for SPB separation.

Regardless of whether Clb/Cdc28 is capable of controlling some aspect of Kip1 and Cin8 stability by regulating APC^Cdh1^ activity, our findings clearly indicate that wild-type Kip1 and Cin8 levels are not sufficient for SPB separation in the absence of Clb/Cdc28 kinase activity (Figure 3 and S2) and are in agreement with those of Robbins & Cross. Thus, we have identified an additional layer of control whereby the direct phosphorylation of kinesin-5 motors is essential for the efficient separation of SPBs and assembly of a short spindle.

It is not fully understood how direct phosphorylation of kinesin-5 motors affects their function in spindle assembly. Although the localization of kinesin-5 motors to the spindle is regulated by cyclin B/Cdk1 phosphorylation in certain metazoan systems [35], [37], [39], [40], we have determined that the localization of Kip1 and Cin8 to the spindle is not dependent on Clb/Cdc28 in budding yeast. Instead, our observations suggest that Clb/Cdc28 phosphorylation regulates some aspect of kinesin-5 motor activity.

Ser 388, the critical consensus CDK site (S/T-P-X-X) we identified in Kip1 is conserved in most known kinesins-5, except for *Schizosaccharomyces pombe* Cut7 in which the orthologous serine is followed by a serine. This site is also the only one conserved between metazoan and fungal kinesins-5. Moreover, sequence comparison and homology modeling both indicate that the structures of the HsEg5 and XlEg5 motor domains bear a greater resemblance to that of Kip1 than that of Cin8. Thus, it is possible that CDK-phosphorylation at the serine orthologous to Ser 388 in Kip1 may be a common regulatory mechanism for spindle assembly in other eukaryotic organisms.

We have also determined that the phosphorylation of Kip1 at two other CDK sites in its C-terminal globular tail domain is also important to its function in promoting SPB separation. It has been reported that the phosphorylation of the BimC box in *Xenopus* Eg5 by cyclin B/Cdk1 enhances binding to microtubules both *in vitro* and in *Xenopus* egg extract [80]. The tail domains of the kinesins-5 are, however, quite divergent, as they are in other kinesin subfamilies [26]. Hence, it remains to be determined if the phosphorylation of the Kip1 tail domain will have the same effect.

Kinesin-5 motors exhibit a variety of functions that could be regulated, including microtubule binding, microtubule crosslinking, ATP binding and hydrolysis, microtubule-based motility or influencing microtubule dynamics [80]–[84]. Furthermore, there have been several reported examples of different aspects of kinesin motor function being regulated through phosphorylation of either their motor or tail domains, including cases where cyclin B/Cdk1 is the kinase involved [80], [85]–[88]. Detailed biochemical studies will be required to dissect the specific kinesin-5 functions controlled by Clb/Cdc28-mediated phosphorylation. Such studies may also reveal a mechanism linking phosphorylation of the kinesin-5 motor domain to that of its tail domain.

## Materials and Methods

### Plasmids and DNA manipulation

Standard methods of DNA manipulation were employed in plasmid construction and PCR. Whenever PCR was involved in gene manipulation, the product was sequenced in full to determine the occurrence of PCR errors. More information on plasmid construction can be found in Text S1.

### Yeast strains and media

All strains are derivatives of BF264-15DU unless otherwise indicated (Table S2). Strains were constructed by standard yeast methods (detailed in Text S1). Yeast cultures were grown in standard YEP medium (1% yeast extract, 2% peptone, 0.012% adenine, 0.006% uracil supplemented with 2% sugar) unless indicated. For synchrony experiments, *bar1* strains were arrested with 25 ng/ml α mating pheromone, also known as α-factor (BioVectra). For experiments involving fluorescence microscopy that used liquid cultures, YEP medium was supplemented with an additional 0.003% adenine (0.015% final).

### Asynchronous temperature shift time course experiments

For experiments involving *cdc4-3* strains, cells were grown in YEP-dextrose (YEPD) medium overnight at 24°C (permissive temperature). Cells were subsequently diluted to a density of 1×10^7^ cells/ml, and then incubated at 24°C for 90 min. Cultures were subsequently shifted to 37°C (restrictive temperature) to inactivate the temperature-sensitive Cdc4-3. Details of the subsequent return to permissive temperature experiment can be found in Text S1.

### Synchronized sugar shift time course experiments

For experiments involving Δ*clb1,2,3,4,5,6* strains, cells were grown in YEP-galactose (YEPG) medium overnight, and then diluted before being allowed to reach a density of 1×10^7^ cells/ml. They were subsequently arrested with α-factor before adding either 20% dextrose to a final concentration of 2% dextrose in order to inhibit the expression of Clb1 in these strains or an equal volume of water to the control. Fifteen minutes after the addition of dextrose/water, cells were released into pre-warmed (30°C) YEPG.

For experiments involving strains with *P_GAL1_-SIC1*Δ*3P* derived from BF264-15DU, cells were grown in non-inducing YEP-sucrose (YEPS) medium overnight and then diluted before being allowed to reach a density of 1×10^7^ cells/ml. They were subsequently arrested with α-factor, then released into pre-warmed (30°C) YEPG. Unlike BF264-15DU strains [89], W303a-derived strains express invertase and can thus hydrolyze sucrose to yield glucose and fructose. Therefore, we determined that YEPS is not a suitable non-inducing medium for W303a-derived strains since they can break sucrose down to form glucose which represses the *GAL1-10* promoter. Instead, these strains had to first be inoculated into filter-sterilized YEPS and grown for a few hours before being shifted to YEP-raffinose (YEPR) for subsequent overnight growth. The cells were arrested with α-factor in YEPR before induction with YEPG.

### Synchronized temperature shift time courses

Cells were grown in YEPD medium overnight at 25°C (permissive temperature) and then diluted and allowed to reach a density of 7.5×10^6^ cells/ml. They were subsequently arrested with α-factor. Arrested cultures were divided in two halves and one half was moved to 37°C for 15 min (restrictive temperature) while the other half remained at 25°C. Cells were then released into YEPD pre-warmed to the respective temperatures.

### Immunoblotting

Cell samples collected during time courses were spun down, washed with ice cold PBS, then frozen in liquid nitrogen. Lysates were prepared by vortexing cells with acid-washed glass beads (Sigma-Aldrich) in modified RIPA buffer (50 mM Tris-HCl pH 7.5, 20 mM Na_4_P_2_O_7_, 250 mM NaCl, 50 mM NaF, 1% NP-40, 2 mM EDTA, 1 mM Na_3_VO_4_, 1 mM DTT, 1.25 mM benzamidine hydrochloride, 0.1 mg/ml PMSF, 1 µg/ml each leupeptin, aprotinin, and pepstatin A). Lysates were cleared by centrifugation at 4°C, and the protein content of the cleared lysates was determined by measuring A_280_ with a Biophotometer (Eppendorf).

Proteins were separated by SDS-PAGE on 8.5% Tris-HCl gels using the Laemmli method and then transferred to Immobilon-P (Millipore) PVDF membranes for antibody probing. Proteins tagged with c-Myc were detected with mouse anti-c-Myc clone 9E10 (Santa Cruz Biotechnology), while mCherry fusions were detected with rabbit anti-DsRed/RFP (MBL). Cdc28 and Pho85 were detected with mouse anti-PSTAIR (Abcam) for use as loading controls. The secondary antibodies used were horseradish peroxidase-conjugated goat anti-mouse (Pierce Thermo-Scientific) and goat anti-rabbit (Abcam). Blots were visualized with Supersignal West Pico chemiluminescent substrate (Pierce Thermo-Scientific).

### Kinase purification for *in vitro* phosphorylation assay

Soluble Clb2/Cdc28 kinase was prepared, using an abbreviated form of the TAP protocol described by Puig and colleagues which excludes the CaM-binding and elution steps [90]. Clb2-TAP was overexpressed from an episomal plasmid, p*GAL-CLB2-TAP* [54], together with Cdc28 in a *swe1*Δ yeast strain (SBY684, a gift from Daniel Lew) to ensure that the purified kinase is not inhibited by Swe1 phosphorylation of Tyr 19 [10]. To provide a negative control, a wild-type strain (SBY1286) carrying a *URA3*-marked episomal plasmid with the *GAL1-10* promoter (YEpUGAL) was used. Both strains were first grown to log phase in synthetic complete dropout medium lacking uracil (SC-Ura) with 2% sucrose, then switched to SC-Ura with 2% galactose. Soluble Clb5/Cdc28 kinase was prepared in a similar manner (see Text S1).

After induction, cells were lysed in modified RIPA buffer by vortexing with glass beads. Clb2/Cdc28 was isolated by binding to IgG-Sepharose beads (Amersham BioSciences/GE Healthcare) followed by overnight cleavage at 4°C with AcTEV protease (Invitrogen) to remove the Protein A portion of the TAP tag. The concentration of Cdc28 was estimated by quantitative western blotting with anti-PSTAIR antibody, using purified GST-Cdk1 (Cell Signaling Technology) as a standard. Densitometric analysis was performed with ImageJ (Wayne Rasband, National Institutes of Health).

### Substrate purification for *in vitro* phosphorylation assay

Kip1-myc_12_, Cin8-myc_12_, and their respective CDK site mutants were overexpressed from episomal plasmids under the control of the *GAL1* promoter in *P_GAL1_-SIC1*Δ*3P* cells. To avoid the formation of tetramers containing the respective endogenous kinesin-5, Kip1-myc12 and Kip1^6A^-myc12 were overexpressed in *kip1*Δ strains (SBY1274, 1276), while Cin8-myc_12_ and Cin8^5A^-myc_12_ were overexpressed in *cin8*Δ strains (SBY1280, 1282). Control strains were included that carry the empty vector alone (SBY1278, 1284). Strains were grown to log phase in synthetic complete dropout medium lacking leucine (SC-Leu) with 2% sucrose, arrested with α-factor, and then released into YEPG.

Cells were lysed in modified RIPA buffer. Immunoprecipitation was carried out by diluting lysate in IP buffer (50 mM Tris-HCl pH 7.5, 300 mM NaCl, 0.1% NP-40, 1 mM EDTA, 0.1 mM DTT, 2.5 mM benzamidine hydrochloride, 0.2 mg/ml PMSF, 2 µg/ml each leupeptin, aprotinin, and pepstatin A) before adding anti-c-Myc IgG agarose beads (Sigma-Aldrich). Binding to the beads was carried out at 4°C for 2 h. Beads were then washed twice with modified RIPA buffer with 500 mM NaCl (instead of 250 mM NaCl), and 0.1 mM DTT added, and twice more with modified RIPA buffer with 0.1% SDS, 0.25% DOC and 0.1 mM DTT added. Finally, beads were washed with 50 mM Tris-HCl, pH 7.5 and divided into smaller portions for the phosphorylation reaction described below. Substrate yield was estimated to be about 0.4 µg of Kip1-myc_12_/Cin8-myc_12_ by performing identical immunoprecipitations for densitometric comparison against BSA (Sigma-Aldrich) standards after SDS-PAGE and staining with Coomassie Brilliant Blue R250.

### *In vitro* phosphorylation assay

Kinesins (∼0.4 µg) immobilized on IgG-agarose beads were washed with kinase reaction buffer (50 mM Tris-HCl pH 7.5, 10 mM MgCl_2_, 25 mM β-glycerophosphate, 1 mM DTT). Subsequently, the beads were mixed with ∼12 ng Clb2/Cdc28 in kinase reaction buffer containing 0.2 mM ATP and 0.5 µCi/µl ^32^P-γ-ATP. One microgram of histone H1 (Roche) was used as a control substrate in a separate reaction. Reactions were incubated at 30°C for 1 h and then halted by adding SDS-PAGE sample buffer before boiling for 5 min. Proteins were separated by electrophoresis on 10% Express PAGE gels (GenScript) and visualized by staining with Coomassie Brilliant Blue R250 (Bio-Rad). Gels were dried and radiolabelling was subsequently detected by autoradiography using a Phosphor Imager and Storm scanner (Molecular Dynamics, GE Healthcare).

### Spot assays for proliferation

Strains were grown overnight at room temperature in YEPD and then diluted to allow resumption of log phase growth at room temperature for at least two hours. A 2×10^6^ cells/ml suspension of each strain was then prepared in YEPD and diluted serially to make 2×10^5^ and 2×10^4^ cells/ml suspensions. Three microliters of each suspension was spotted on YEPD plates and the plates were subsequently incubated at either ambient room temperature (∼22°C) or 37°C, as indicated. Plates were imaged using a Bio-Rad GelDoc and the software QuantityOne (Bio-Rad).

### Flow cytometry

Cells were prepared for flow cytometric analysis of DNA content using SYTOX Green (Invitrogen) as previously described [91]. Graphs used in figures were generated using WinMDI 2.9 (Scripps Research Institute).

### Fluorescence microscopy

For fluorescence microscopy, cells growing in liquid medium were sonicated, then spun down and fixed in 2.0–2.5% paraformaldehyde at room temperature for 5 min, then washed twice with PBS. Cells growing on fresh plates no more than two days old were simply resuspended in water on glass slides before imaging. All fluorescence microscopy was performed on a Zeiss Axio Imager widefield fluorescence microscope controlled with MetaMorph 7.5 (Molecular Devices, MDS Analytical Technologies). Images were captured with a Hamamatsu Orca ER monochrome cooled-CCD camera and analyzed with both MetaMorph 7.5 and Adobe Photoshop 7.0 (Adobe Labs).

### Mass spectrometry analysis of protein phosphorylation

Kip1-myc_12_ and Cin8-myc_12_ were overexpressed in yeast in a similar manner to that described above. Additionally, the two kinesins-5 were overexpressed in strains that also overexpress either Sic1Δ3P to inhibit Clb/Cdc28 kinase activity or Clb2-HA_3_ to increase the *in vivo* phosphorylation of Clb/Cdc28 substrates. Cells were lysed and the myc-tagged proteins were immunopurified as described above, except using different buffers that contained a high concentration of phosphatase inhibitors to better preserve phosphorylated amino acid residues. The anti-phosphatase lysis buffer used had the following composition: 50 mM Tris-HCl pH 7.5, 500 mM NaCl, 20 mM Na_4_P_2_O_7_, 150 mM NaF, 150 mM β-glycerophosphate, 2mM Na_3_VO_4_, 1 mM EDTA, 1 mM EGTA, 1% NP-40, 1 mM DTT, 1.25 mM benzamidine hydrochloride, 1 mM PMSF, 1 µg/ml each leupeptin, aprotinin, and pepstatin A. The anti-phosphatase IP buffer used had the following composition: 50 mM Tris-HCl pH 7.5, 300 mM NaCl, 200 mM NaF, 200 mM β-glycerophosphate, 2mM Na_3_VO_4_, 1 mM EDTA, 0.1% NP-40, 0.1 mM DTT, 2 mM PMSF, 2 µg/ml each leupeptin, aprotinin, and pepstatin A. Following immunoprecipitation, the anti-c-Myc IgG agarose beads were boiled in SDS-PAGE sample buffer and the liberated proteins separated by SDS-PAGE. Gels were fixed and then stained with Coomassie Brilliant Blue R250. Bands corresponding to Kip1-myc_12_ and Cin8-myc_12_ were excised and sent to the Taplin Mass Spectrometry Facility at Harvard University for analysis.

Excised gel bands were cut into approximately 1 mm^3^ pieces. The samples were reduced with 1 mM DTT for 30 minutes at 60°C and then alkylated with 5mM iodoacetamide for 15 minutes in the dark at room temperature. Gel pieces were then subjected to a modified in-gel trypsin digestion procedure [92]. Gel pieces were washed and dehydrated with acetonitrile for 10 min followed by the removal of acetonitrile. Pieces were then completely dried in a speed-vac before rehydration with 50 mM ammonium bicarbonate solution containing 12.5 ng/µl modified sequencing-grade trypsin (Promega, Madison, WI) at 4°C. Samples were then placed in a 37°C room overnight. Peptides were later extracted by removing the ammonium bicarbonate solution, followed by one wash with a solution containing 50% acetonitrile and 5% acetic acid. The extracts were then dried in a speed-vac (∼1 h). The samples were then stored at 4°C until analysis.

On the day of analysis, the samples were reconstituted in 5 µl of HPLC solvent A (2.5% acetonitrile, 0.1% formic acid). A nano-scale reverse-phase HPLC capillary column was created by packing 5 µm C18 spherical silica beads into a fused silica capillary (100 µm inner diameter×12 cm length) with a flame-drawn tip [93]. After equilibrating the column, each sample was pressure-loaded off-line onto the column. The column was then reattached to the HPLC system. A gradient was formed and peptides were eluted with increasing concentrations of solvent B (97.5% acetonitrile, 0.1% formic acid).

As each peptide was eluted, it was subjected to electrospray ionization, and the resulting ions entered a LTQ-Orbitrap mass spectrometer (ThermoFinnigan, San Jose, CA). Eluting peptides were detected, isolated, and fragmented to produce a tandem mass spectrum of specific fragment ions for each peptide. Peptide sequences were determined by matching protein or translated nucleotide databases with the acquired fragmentation pattern by the software program, Sequest (ThermoFinnigan, San Jose, CA) [94]. The modification of 79.9663 mass units to serine, threonine, and tyrosine was included in the database searches to determine phosphopeptides. Each phosphopeptide that was determined by the Sequest program was also manually inspected to ensure confidence.

### Homology modeling

Homology models were constructed using DeepView Swiss PDB Viewer (v4.0) and the Swiss MODEL web server [64]–[66]. Initial amino acid sequence alignments for the Kip1 and Cin8 motor domains were performed using CLUSTALW through the software BioEdit (Tom Hall, Ibis BioSciences) against the primary sequences of the HsEg5/Kif11 and Kar3 motor domains. Initial model construction was then done in DeepView against a solved crystal structure for the HsEg5 motor domain (ExPDB 1ii6B, the base template). Additional template structures (ExPDB 1ii6A for HsEg5 and 3kar_ for Kar3) were subsequently aligned against the base template and the project submitted to Swiss MODEL. The initial models were examined for structural errors, particularly in loop placement. The errors identified were used to adjust the sequence alignments and repeat the modeling process until stable structures with a negative total energy mostly free of backbone problems were obtained. Model quality was evaluated with What Check [95] and DeepView was used to fix side chain errors identified. Residue swaps were performed using the “Mutate” function and the rotamer library included in Swiss PDB Viewer [96]. Images were rendered with Swiss PDB Viewer and Persistence of Vision Raytracer v3.6 (Persistence of Vision Pty. Ltd.).

## Supporting Information

Figure S1 Proliferation of *cdc4-3*(ts) *sic1*Δ cells at permissive temperature following arrest at the restrictive temperature. (A) Spot assay comparing the proliferation of *cdc4-3 sic1*Δ cells that were previously grown in liquid culture at 37°C for the indicated amount of time (2, 4 or 6 h). For comparison, *cdc4-3 SIC1* and *cdc4-3 sic1*Δ cells that were not exposed to the restrictive temperature (0 h) were also spotted. Prior to spotting, strains were grown in liquid culture to log phase at 24°C before being shifted to 37°C (see text, Figure 1). For spotting, strains were diluted to 2×10^6^ cells/ml, and then further diluted serially to 2×10^4^ cells/ml. An equal volume of cells from each dilution was spotted on YEPD, and plates were incubated at ambient temperature (∼22°C); plates were imaged 50 and 61h after spotting. (B) Micrographs showing the morphology and SPBs (marked with Spc42-GFP) of live *cdc4-3 sic1*Δ cells growing on plate media following their return to the permissive temperature. Scale bar: 2 µm.
(0.96 MB TIF)

Figure S2 Phosphorylation of Kip1 and Cin8 by Clb5/Cdc28 *in vitro*. Wild-type Kip1 and Cin8, as well as their multiple consensus CDK site mutant forms (Kip1^6A^, Cin8^5A^) were immunoprecipitated from yeast lysates and mixed with soluble Clb5/Cdc28, also prepared from yeast, and ^32^P-γ-ATP. Soluble histone H1 (1.0 µg) was used as a control substrate. Proteins were subjected to SDS-PAGE after one hour at 30°C. PhosphorImages are shown on top and corresponding Coomassie-stained bands below. Unmarked lanes either contain molecular weight standards or had no protein loaded.
(0.17 MB TIF)

Figure S3 DNA replication and SPB separation. In (A) *KIP1-12MYC* and (B) *CIN8-12MYC* strains in the presence and the absence of active Clb/Cdc28 kinase. Data were collected from cultures used in the experiments detailed in Figure 3. Strains that carry the *P_GAL1_-SIC1*Δ*3P* transgene are indicated and control strains that do not are indicated as “wild-type”. Cells were first synchronized in G1 with α-factor before being released into galactose medium; cells were fixed at the indicated times for flow cytometric analysis and SPB counts. Histograms derived from flow cytometry show DNA content on the horizontal axis and number of counts on the vertical axis. SPB counts are presented as white bars for *P_GAL1_-SIC1*Δ*3P* strains and black bars for control strains.
(0.33 MB TIF)

Figure S4 Impaired proliferation of strains with CDK site point mutants (Ser/Thr→Ala) of either (A) Kip1 or (B) Cin8 as their only kinesin-5. Strains growing in log phase at permissive temperature were diluted to 2×10^6^ cells/ml, and then further diluted serially to 2×10^4^ cells/ml. An equal volume of cells from each dilution was spotted on YEPD, and plates were incubated at either ambient temperature (∼22°C) or 37°C. The number above each column of spots indicates the cell density (cells/ml). All alleles compared were untagged to control for the effects of the mCherry fusion in the strains shown in Figure 5.
(1.12 MB TIF)

Figure S5 Abundance and localization of Kip1^S388A^ and Kip1^S1037A, T1040A^ compared with that of wild-type Kip1 at 37°C. (A) *CIN8 kip1*Δ cells expressing either Kip1-mCherry (♦), Kip1^S388A^-mCherry or Kip1^S1037A, T1040A^-mCherry (○) integrated under the control of the *KIP1* promoter were arrested with α-factor, and then released at 37°C. The abundance of each mCherry fusion protein was determined by western blotting with anti-RFP/DsRed. Anti-PSTAIR was used as a loading control. (B) Fluorescence images of the same *CIN8 kip1*Δ strains at 37°C, 60 min after being released from α-factor arrest. Scale bar: 2 µm.
(0.70 MB TIF)

Figure S6 Flow cytometric analysis of asynchronous populations of yeast strains bearing Kip1 or Cin8 CDK mutant alleles as their only source of kinesin-5. Cells were grown in liquid culture to log phase at ambient room temperature (∼22°C) before being shifted to 37°C for 3 h. Histograms show DNA content on the horizontal axis and counts on the vertical axis. *KIP1* (A) and *CIN8* (B) allele combinations are indicated. Bar graphs are shown at the bottom indicating the relative proportions of cells having 1C (M1, spotted bars) and 2C DNA (M2, black bars) for each strain.
(0.11 MB TIF)

Table S1 Peak intensities measured for phosphopeptides and their corresponding unphosphorylated forms identified during LC/MS/MS analysis of Kip1-myc_12_ and Cin8-myc_12_. Confidently assigned phosphorylation sites are indicated with an asterisk (*) while phosphorylation site assigned with less confidence are indicated with a number sign/hash (#); the latter are the most likely sites on the respective phosphopeptides and were verified through repeated observation and confident assignments in other phosphopeptides or in other strains. Candidate kinases for each assigned phosphorylation site are also listed.
(0.02 MB XLS)

Table S2 Yeast strains used in this study. All strains are derivatives of BF264-15DU unless otherwise indicated.
(0.12 MB DOC)

Text S1 Supporting materials and methods.
(0.06 MB DOC)
