# Supplementary figures and images for: B-Cyclin/CDKs Regulate Mitotic Spindle Assembly by Phosphorylating Kinesins-5 in Budding Yeast
Source: PLoS Genet. 2010 May 6;6(5):e1000935. doi: 10.1371/journal.pgen.1000935 (PMC2865516; doi:10.1371/journal.pgen.1000935)

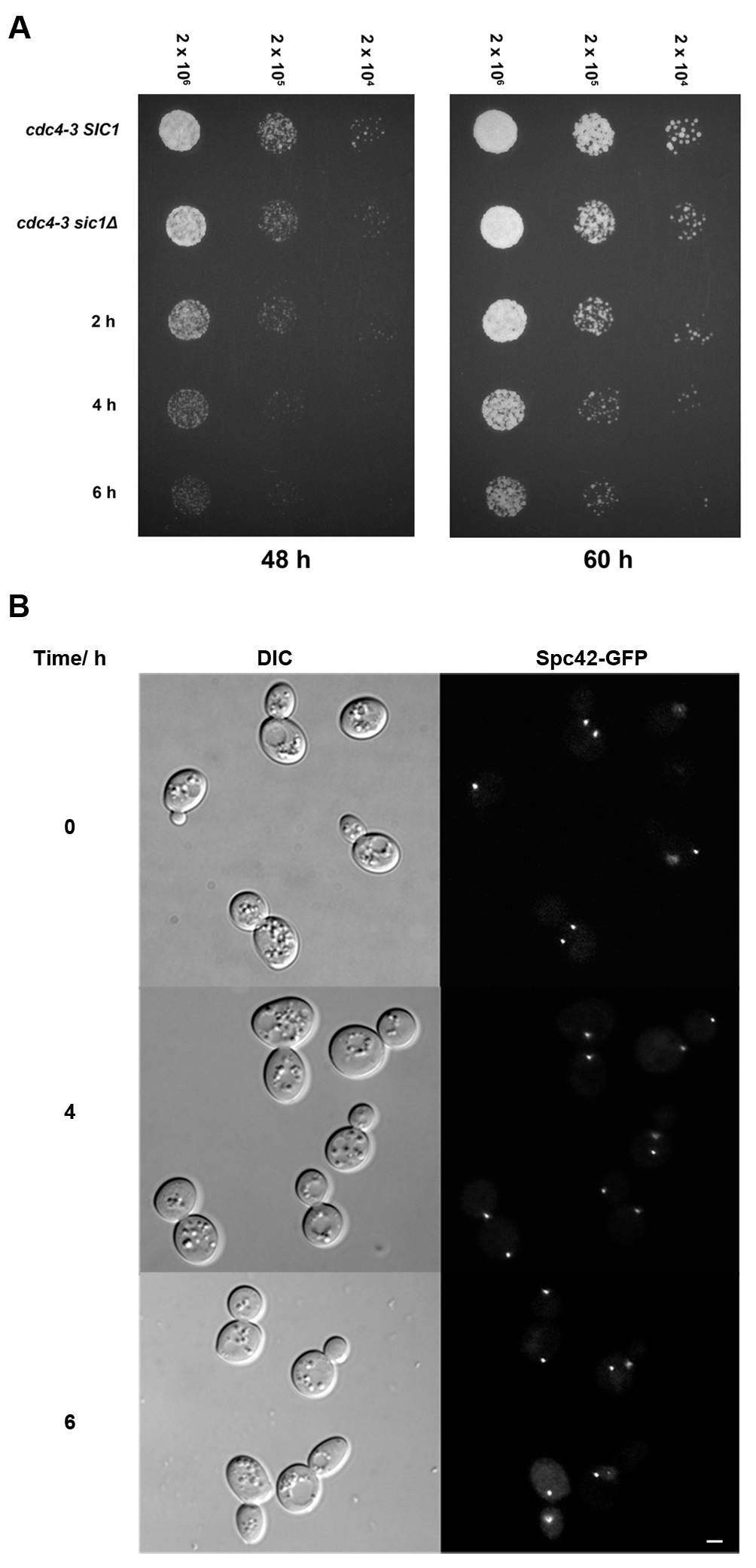

Supplement: Figure S1 — Proliferation of cdc4-3(ts) sic1Δ cells at permissive temperature following arrest at the restrictive temperature. (A) Spot assay comparing the proliferation of cdc4-3 sic1Δ cells that were previously grown in liquid culture at 37°C for the indicated amount of time (2, 4 or 6 h). For comparison, cdc4-3 SIC1 and cdc4-3 sic1Δ cells that were not exposed to the restrictive temperature (0 h) were also spotted. Prior to spotting, strains were grown in liquid culture to log phase at 24°C before being shifted to 37°C (see text, Figure 1). For spotting, strains were diluted to 2×10^6^ cells/ml, and then further diluted serially to 2×10^4^ cells/ml. An equal volume of cells from each dilution was spotted on YEPD, and plates were incubated at ambient temperature (∼22°C); plates were imaged 50 and 61h after spotting. (B) Micrographs showing the morphology and SPBs (marked with Spc42-GFP) of live cdc4-3 sic1Δ cells growing on plate media following their return to the permissive temperature. Scale bar: 2 µm. (0.96 MB TIF) [file pgen.1000935.s001.tif]

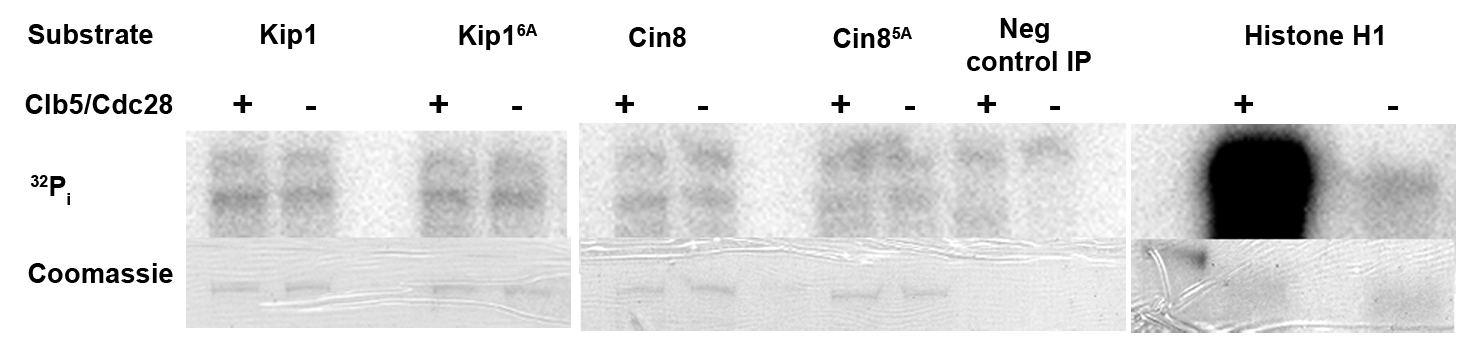

Supplement: Figure S2 — Phosphorylation of Kip1 and Cin8 by Clb5/Cdc28 in vitro. Wild-type Kip1 and Cin8, as well as their multiple consensus CDK site mutant forms (Kip16A, Cin85A) were immunoprecipitated from yeast lysates and mixed with soluble Clb5/Cdc28, also prepared from yeast, and 32P-γ-ATP. Soluble histone H1 (1.0 µg) was used as a control substrate. Proteins were subjected to SDS-PAGE after one hour at 30°C. PhosphorImages are shown on top and corresponding Coomassie-stained bands below. Unmarked lanes either contain molecular weight standards or had no protein loaded. (0.17 MB TIF) [file pgen.1000935.s002.tif]

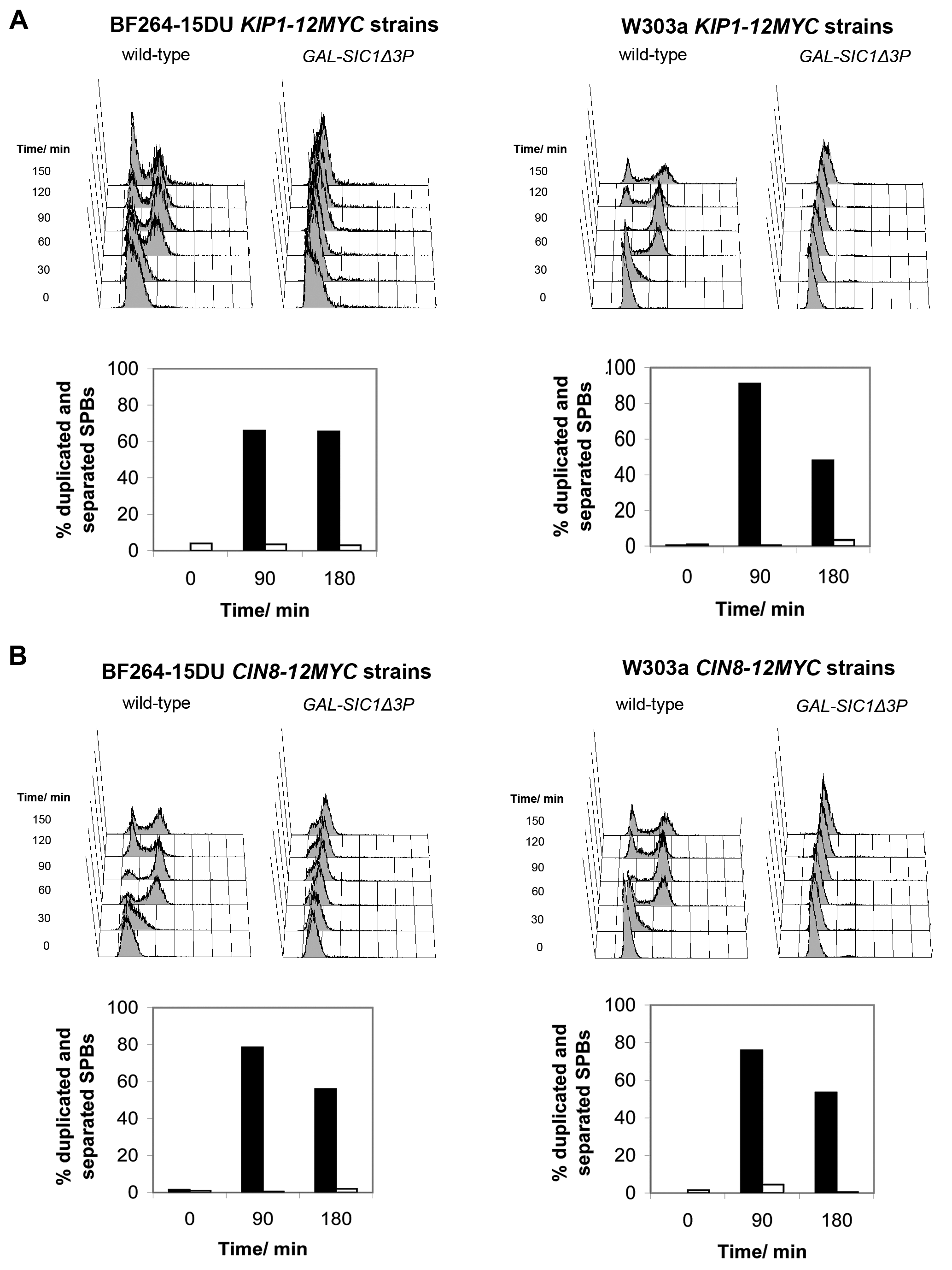

Supplement: Figure S3 — DNA replication and SPB separation. In (A) KIP1-12MYC and (B) CIN8-12MYC strains in the presence and the absence of active Clb/Cdc28 kinase. Data were collected from cultures used in the experiments detailed in Figure 3. Strains that carry the PGAL1-SIC1Δ3P transgene are indicated and control strains that do not are indicated as “wild-type”. Cells were first synchronized in G1 with α-factor before being released into galactose medium; cells were fixed at the indicated times for flow cytometric analysis and SPB counts. Histograms derived from flow cytometry show DNA content on the horizontal axis and number of counts on the vertical axis. SPB counts are presented as white bars for PGAL1-SIC1Δ3P strains and black bars for control strains. (0.33 MB TIF) [file pgen.1000935.s003.tif]

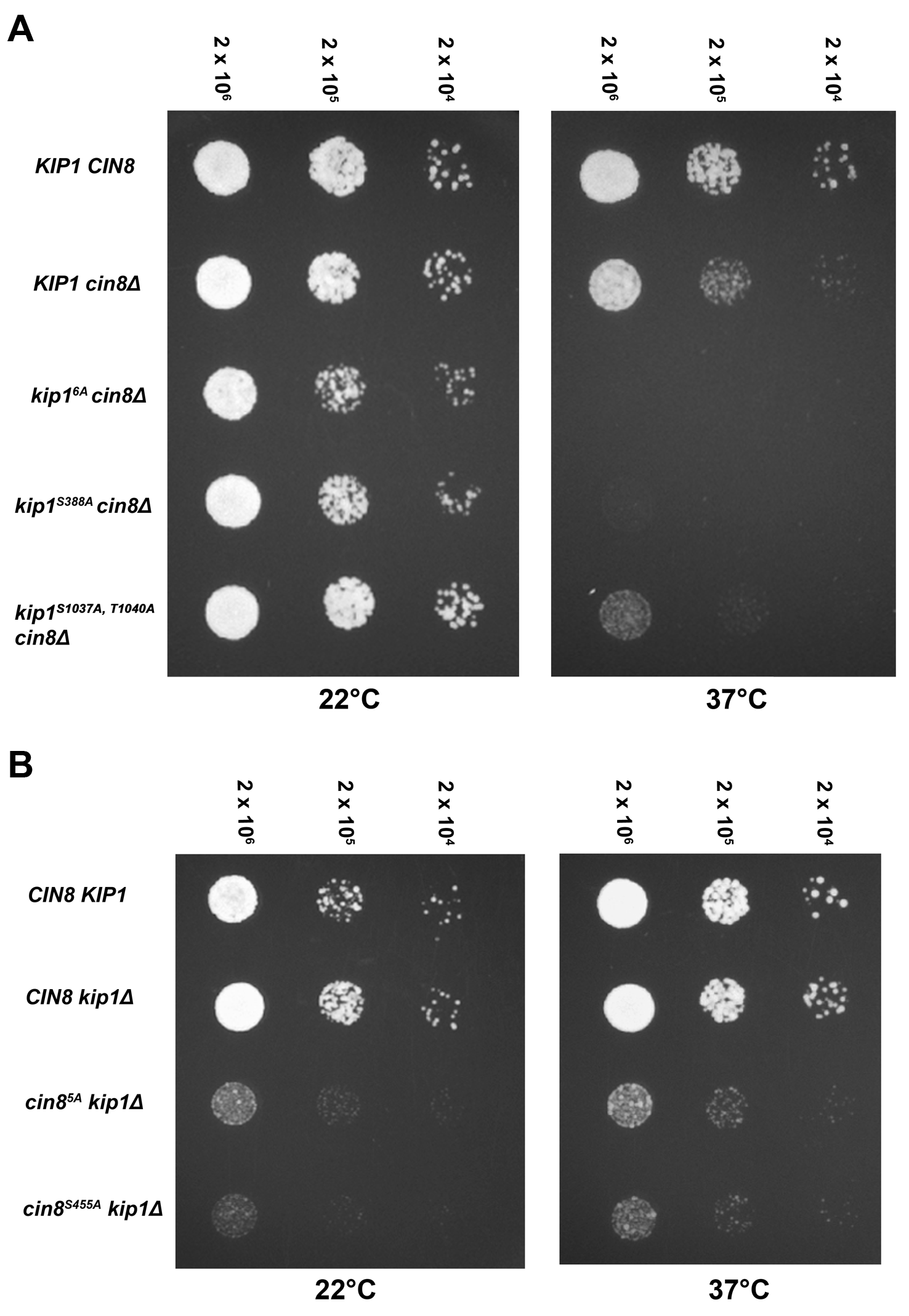

Supplement: Figure S4 — Impaired proliferation of strains with CDK site point mutants (Ser/Thr→Ala) of either (A) Kip1 or (B) Cin8 as their only kinesin-5. Strains growing in log phase at permissive temperature were diluted to 2×10^6^ cells/ml, and then further diluted serially to 2×10^4^ cells/ml. An equal volume of cells from each dilution was spotted on YEPD, and plates were incubated at either ambient temperature (∼22°C) or 37°C. The number above each column of spots indicates the cell density (cells/ml). All alleles compared were untagged to control for the effects of the mCherry fusion in the strains shown in Figure 5. (1.12 MB TIF) [file pgen.1000935.s004.tif]

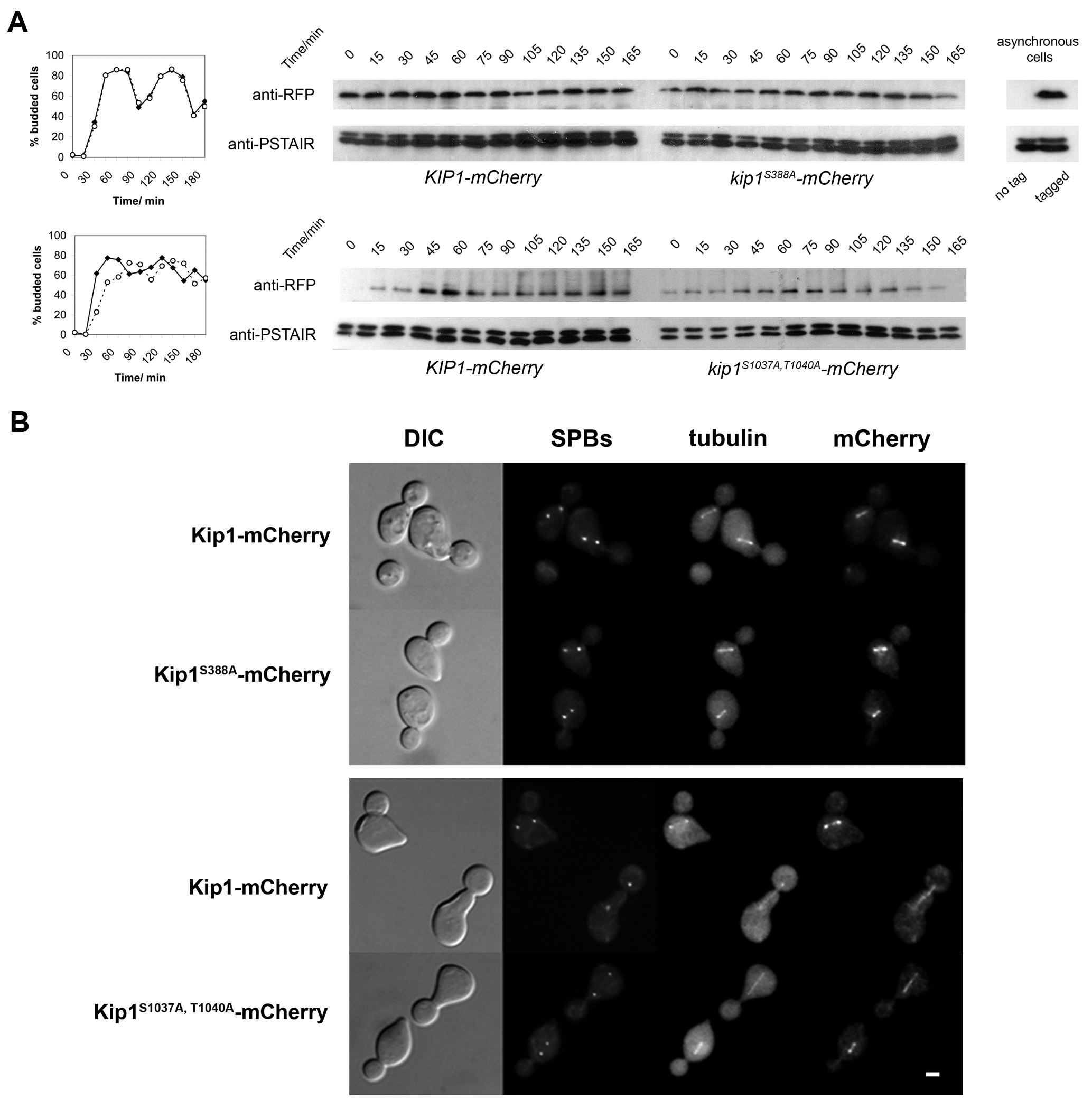

Supplement: Figure S5 — Abundance and localization of Kip1S388A and Kip1S1037A, T1040A compared with that of wild-type Kip1 at 37°C. (A) CIN8 kip1Δ cells expressing either Kip1-mCherry (♦), Kip1S388A-mCherry or Kip1S1037A, T1040A-mCherry (○) integrated under the control of the KIP1 promoter were arrested with α-factor, and then released at 37°C. The abundance of each mCherry fusion protein was determined by western blotting with anti-RFP/DsRed. Anti-PSTAIR was used as a loading control. (B) Fluorescence images of the same CIN8 kip1Δ strains at 37°C, 60 min after being released from α-factor arrest. Scale bar: 2 µm. (0.70 MB TIF) [file pgen.1000935.s005.tif]

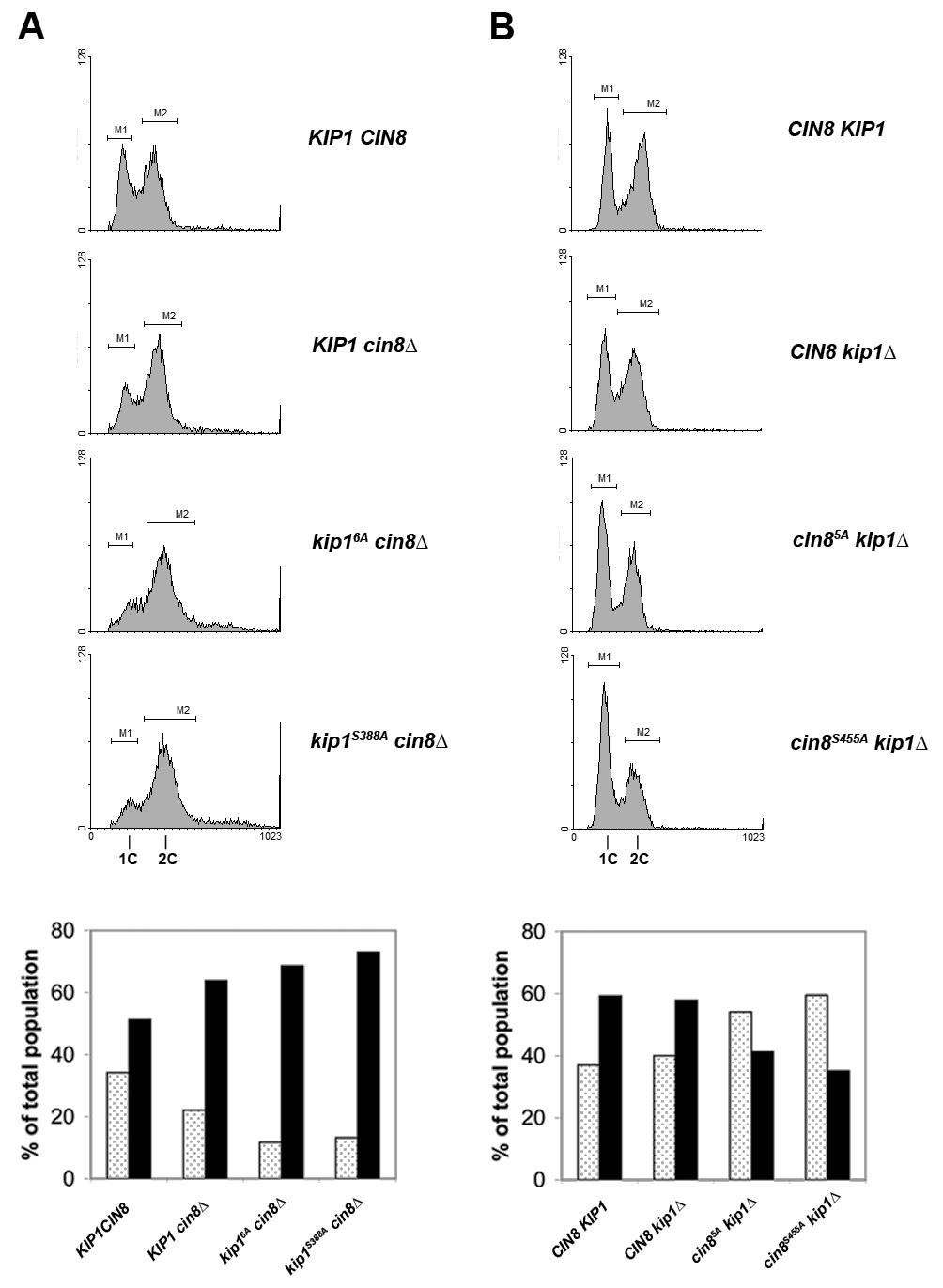

Supplement: Figure S6 — Flow cytometric analysis of asynchronous populations of yeast strains bearing Kip1 or Cin8 CDK mutant alleles as their only source of kinesin-5. Cells were grown in liquid culture to log phase at ambient room temperature (∼22°C) before being shifted to 37°C for 3 h. Histograms show DNA content on the horizontal axis and counts on the vertical axis. KIP1 (A) and CIN8 (B) allele combinations are indicated. Bar graphs are shown at the bottom indicating the relative proportions of cells having 1C (M1, spotted bars) and 2C DNA (M2, black bars) for each strain. (0.11 MB TIF) [file pgen.1000935.s006.tif]
