# Supplementary material for: B-Cyclin/CDKs Regulate Mitotic Spindle Assembly by Phosphorylating Kinesins-5 in Budding Yeast
Source: PLoS Genet. 2010 May 6;6(5):e1000935. doi: 10.1371/journal.pgen.1000935 (PMC2865516; doi:10.1371/journal.pgen.1000935)
Supplement: Table S2 — Yeast strains used in this study. All strains are derivatives of BF264-15DU unless otherwise indicated. (0.12 MB DOC) [file pgen.1000935.s008.doc]

**Table S2.** **Yeast strains used in this study.**

All strains are derivatives of BF264-15DU unless otherwise indicated.

| **Strain** | **Genotype** | **Source** |
| --- | --- | --- |
| SBY408 | *MATa bar1 SPC42-GFP::TRP1::zeoR* | Haase et al., 2001 |
| SBY621 | *MATa bar1 SPC42-GFP::TRP1::zeoR CIN8-12MYC::kanMX2* | This study |
| SBY627 | *MATa bar1 SPC42-GFP::TRP1::zeoR P_GAL1_-SIC1*Δ*3P::URA3 CIN8-12MYC::kanMX2* | This study |
| SBY680 | *MATa bar1 SPC42-GFP::TRP1::zeoR* YEpLGAL | This study |
| SBY684 | *MATa bar1 swe1::LEU2 P_GAL1_-CDC28::TRP1* p*GAL-CLB2-TAP* | DJ Lew |
| SBY748 | *MATa bar1 SPC42-GFP::TRP1::zeoR P_GAL1_-SIC1*∆*3P::URA3* YEpLGAL*-CIN8-12MYC* | This study |
| SBY750 | *MATa bar1 SPC42-GFP::TRP1::zeoR* YEpLGAL*-CIN8-12MYC* | This study |
| SBY789 | *MATa bar1 SPC42-GFP::TRP1::zeoR P_GAL1_-SIC1*∆*3P::URA3* YEpLGAL*-KIP1-12MYC* | This study |
| SBY791 | *MATa bar1 SPC42-GFP::TRP1::zeoR* YEpLGAL*-KIP1-12MYC* | This study |
| SBY866 | *MATa bar1 SPC42-GFP::TRP1::zeoR cin8*Δ*::LEU2* | This study |
| SBY934 | *MATa bar1::hphMX4 clb1::URA3 clb2::LEU2 clb3::TRP1 clb4::HIS2 clb5::ARG4 clb6::ADE1 P_GAL1_-CLB1::LEU2* | This study |
| SBY938 | *MATa bar1::hphMX4 clb1::URA3 clb2::LEU2 clb3::TRP1 clb4::HIS2 clb5::ARG4 clb6::ADE1 P_GAL1_-CLB1::LEU2 CIN8-12MYC::kanMX2* | This study |
| SBY954 | *MATa bar1 SPC42-GFP::TRP1::zeoR kip1*Δ*::kanMX4* | This study |
| SBY960 | *MATa bar1 SPC42-GFP::TRP1::zeoR CIN8-mCherry::kanMX2 P_HIS3_-CFP-TUB1::URA3* | This study |
| SBY962 | *MATa bar1 SPC42-GFP::TRP1::zeoR CIN8-mCherry::kanMX2 P_GAL1_-SIC1*Δ*3P::HIS2 P_HIS3_-CFP-TUB1::URA3* | This study |
| SBY966 | *MATa bar1 SPC42-GFP::TRP1::zeoR cin8*Δ*::LEU2 cin8^5A^-mCherry::HIS2 P_HIS3_-CFP-TUB1::URA3* | This study |
| SBY972 | *MATa bar1 SPC42-GFP::TRP1::zeoR cin8*Δ*::LEU2 cin8^5A^-mCherry::HIS2 P_HIS3_-CFP-TUB1::URA3 kip1*Δ*::hph4* | This study |
| SBY974 | *MATa bar1 cdc4-3 SPC42-GFP::TRP1* | This study |
| SBY980 | *MATa bar1 SPC42-GFP::TRP1::zeoR cin8*Δ*::LEU2 CIN8-mCherry::HIS2 P_HIS3_-CFP-TUB1::URA3* | This study |
| SBY984 | *MATa bar1 SPC42-GFP::TRP1::zeoR cin8*Δ*::LEU2 CIN8-mCherry::HIS2 P_HIS3_-CFP-TUB1::URA3 kip1*Δ*::hphMX4* | This study |
| SBY991 | *MATa bar1 SPC42-GFP::TRP1::zeoR KIP1-12MYC::kanMX2* | This study |
| SBY993 | *MATa bar1 SPC42-GFP::TRP1::zeoR P_GAL1_-SIC1*Δ*3P::HIS2 KIP1-12MYC::kanMX2* | This study |
| SBY1000 | *MATa bar1 cdc4-3 SPC42-GFP::TRP1 sic1::URA3* | This study |
| SBY1038 | *MATa bar1 SPC42-GFP::TRP1::zeoR kip1*Δ*::kanMX4 KIP1-mCherry::LEU2 P_HIS3_-CFP-TUB1::URA3* | This study |
| SBY1040 | *MATa bar1 SPC42-GFP::TRP1::zeoR kip1*Δ*::kanMX4 kip1^6A^-mCherry::LEU2 P_HIS3_-CFP-TUB1::URA3* | This study |
| SBY1064 | *MATa bar1 SPC42-GFP::TRP1::zeoR kip1*Δ*::kanMX4 KIP1-mCherry::LEU2 P_HIS3_-CFP-TUB1::URA3 cin8*Δ*::hphMX4* | This study |
| SBY1066 | *MATa bar1 SPC42-GFP::TRP1::zeoR kip1*Δ*::kanMX4 kip1^6A^-mCherry::LEU2 P_HIS3_-CFP-TUB1::URA3 cin8*Δ*::hphMX4* | This study |
| SBY1096 | *MATa bar1 SPC42-GFP::TRP1::zeoR kip1*Δ*::kanMX4 P_HIS3_-CFP-TUB1::URA3* | This study |
| SBY1123 | *MATa bar1 SPC42-GFP::TRP1::zeoR kip1*Δ*::kanMX4 P_HIS3_-CFP-TUB1::URA3 kip1^S388A^-mCherry::LEU2* | This study |
| SBY1129 | *MAT a bar1 SPC42-GFP::TRP1::zeoR kip1*Δ*::kanMX4 P_HIS3_-CFP-TUB1::URA3 kip1^S1037A,T1040A^-mCherry::LEU2* | This study |
| SBY1134 | *MATa bar1 SPC42-GFP::TRP1::zeoR KIP1-mCherry::kanMX2 P_HIS3_-CFP-TUB1::URA3* | This study |
| SBY1135 | *MATa bar1 SPC42-GFP::TRP1::zeoR P_GAL1_-SIC1*Δ*3P::HIS2 KIP1-mCherry::kanMX2 P_HIS3_-CFP-TUB1::URA3* | This study |
| SBY1143 | *MATa bar1 SPC42-GFP::TRP1::zeoR kip1*Δ*::kanMX4 KIP1::URA3* | This study |
| SBY1147 | *MAT a bar1 SPC42-GFP::TRP1::zeoR kip1*Δ*::kanMX4 P_HIS3_-CFP-TUB1::URA3 kip1^S388A^-mCherry::LEU2 cin8*Δ*::hphMX4* | This study |
| SBY1153 | *MATa bar1 SPC42-GFP::TRP1::zeoR kip1*Δ*::kanMX4 KIP1::URA3 cin8*Δ*::hphMX4* | This study |
| SBY1155 | *MATa bar1 SPC42-GFP::TRP1::zeoR kip1*Δ*::kanMX4 kip1^6A^::URA3 cin8*Δ*::hphMX4* | This study |
| SBY1160 | *MAT a bar1::hphMX4 clb1::URA3 clb2::LEU2 clb3::TRP1 clb4::HIS2 clb5::ARG4 clb6::ADE1 P_GAL1_-CLB1::LEU2 KIP1-12MYC::kanMX2* | This study |
| SBY1188 | W303a *MAT a bar1 SPC42-GFP::TRP1::zeoR* | This study |
| SBY1191 | W303a *MATa bar1 SPC42-GFP::TRP1::zeoR KIP1-12MYC::kanMX2* | This study |
| SBY1193 | W303a *MATa bar1 SPC42-GFP::TRP1::zeoR KIP1-12MYC::kanMX2 P_GAL1_-SIC1*Δ*3P::URA3* | This study |
| SBY1219 | *MATa bar1 SPC42-GFP::TRP1::zeoR kip1*Δ*::kanMX4 kip1^S455A^::URA3 cin8*Δ*::hphMX4* | This study |
| SBY1223 | W303a *MATa bar1 SPC42-GFP::TRP1::zeoR CIN8-12MYC::kanMX2* | This study |
| SBY1225 | W303a *MATa bar1 SPC42-GFP::TRP1::zeoR P_GAL1_-SIC1*Δ*3P::URA3 CIN8-12MYC::kanMX2* | This study |
| SBY1274 | *MATa bar1 SPC42-GFP::TRP1::zeoR cin8*Δ*::kanMX4 P_GAL1_-SIC1*Δ*3P::HIS2* YEpLGAL-*CIN8-12MYC* | This study |
| SBY1276 | *MATa bar1 SPC42-GFP::TRP1::zeoR cin8*Δ*::kanMX4 P_GAL1_-SIC1*Δ*3P::HIS2* YEpLGAL-*cin8^5A^-12MYC* | This study |
| SBY1278 | *MATa bar1 SPC42-GFP::TRP1::zeoR cin8*Δ*::kanMX4 P_GAL1_-SIC1*Δ*3P::HIS2* YEpLGAL | This study |
| SBY1280 | *MATa bar1 SPC42-GFP::TRP1::zeoR kip1*Δ*::kanMX4 P_GAL1_-SIC1*Δ*3P::HIS2* YEpLGAL-*KIP1-12MYC* | This study |
| SBY1282 | *MATa bar1 SPC42-GFP::TRP1::zeoR kip1*Δ*::kanMX4 P_GAL1_-SIC1*Δ*3P::HIS2* YEpLGAL-*kip1^6A^-12MYC* | This study |
| SBY1284 | *MATa bar1 SPC42-GFP::TRP1::zeoR kip1*Δ*::kanMX4 P_GAL1_-SIC1*Δ*3P::HIS2* YEpLGAL | This study |
| SBY1286 | *MATa bar1* YEpUGAL | This study |
| SBY1327 | *MATa bar1 SPC42-GFP::TRP1::zeoR P_HIS3_-CFP-TUB1::URA3 cin8*Δ*::LEU2 P_CIN8_-cin8^S455A^-mCherry::HIS2* | This study |
| SBY1350 | *MATa bar1 SPC42-GFP::TRP1::zeoR P_HIS3_-CFP-TUB1::URA3 cin8*Δ*::LEU2 P_CIN8_-cin8^S455A^-mCherry::HIS2 kip1*Δ*::hphMX4* | This study |
| SBY1352 | *MATa bar1 swe1::LEU2 P_GAL1_-CDC28::TRP1* p*GAL-CLB5-TAP::URA3* | DJ Lew |
| SBY1355 | *MATa bar1 SPC42-GFP::TRP1::zeoR cin8*Δ*::LEU2 CIN8::URA3* | This study |
| SBY1365 | *MATa bar1 SPC42-GFP::TRP1::zeoR cin8*Δ*::LEU2 CIN8::URA3 kip1*Δ*::hphMX4* | This study |
| SBY1367 | *MATa bar1 SPC42-GFP::TRP1::zeoR cin8*Δ*::LEU2 cin8^5A^::URA3 kip1*Δ*::hphMX4* | This study |
| SBY1371 | *MATa bar1 SPC42-GFP::TRP1::zeoR cin8*Δ*::LEU2 cin8^S455A^::URA3 kip1*Δ*::hphMX4* | This study |
| SBY1373 | *MATa bar1::hphMX4 clb1::URA3 clb2::LEU2 clb3::TRP1 clb4::HIS2 clb5::ARG4 clb6::ADE1 P_GAL1_-CLB1::LEU2 KIP1-mCherry::kanMX4 SPC42-GFP::TRP1::natMX4* | This study |
| SBY1375 | *MATa bar1::hphMX4 clb1::URA3 clb2::LEU2 clb3::TRP1 clb4::HIS2 clb5::ARG4 clb6::ADE1 P_GAL1_-CLB1::LEU2 CIN8-mCherry::kanMX4 SPC42-GFP::TRP1::natMX4* | This study |
| SBY1388 | *MATa bar1 SPC42-GFP::TRP1::zeoR P_HIS3_-CFP-TUB1::URA3 cin8*Δ*::LEU2 P_CIN8_-cin8^5A^-mCherry::HIS2 kip1*Δ*::hphMX4* | This study |
| SBY1578 | *MATa bar1* YEpGAL*-CLB2-3HA::TRP1 pep4::URA3* YEpLGAL-*KIP1-12MYC* | This study |
| SBY1580 | *MATa bar1* YEpGAL*-CLB2-3HA::TRP1 pep4::URA3* YEpLGAL-*CIN8*-*12MYC* | This study |
| SBY1582 | *MATa bar1* YEpGAL*-CLB2-3HA::TRP1 pep4::URA3* YEpLGAL | This study |
