# Supplementary material for: B-Cyclin/CDKs Regulate Mitotic Spindle Assembly by Phosphorylating Kinesins-5 in Budding Yeast
Source: PLoS Genet. 2010 May 6;6(5):e1000935. doi: 10.1371/journal.pgen.1000935 (PMC2865516; doi:10.1371/journal.pgen.1000935)
Supplement: Text S1 — Supporting materials and methods. (0.06 MB DOC) [file pgen.1000935.s009.doc]

**Supporting Materials and Methods**

**Plasmid construction.** *KIP1* and *CIN8* were amplified from *S. cerevisiae* (BF264-15Dau) genomic DNA by PCR and TA cloned into pDrive (Qiagen). The *CIN8* translation start used is the same as that in the current sequence annotation available on the Saccharomyces Genome Database and not the one located 114 nucleotides upstream that was originally identified by Hoyt et al. [1]; the CIN8 annotation was last updated on 9/22/2003 based on an analysis of closely related yeast species. Both genes were amplified with an optimal Kozak sequence (AAA) preceding the start codon (ATG) to facilitate overexpression in yeast. Point mutations were introduced using site-directed PCR mutagenesis with either *Pfu* (Stratagene) or KOD HotStart DNA polymerase (Toyobo/Novagen). For generating C-terminal fusions, the c-Myc epitope tag was PCR-amplified from pFA6a-*12MYC*-*kanMX2* (a gift from Peter Kaiser) and mCherry was PCR-amplified from pRSET-B-mCherry [2]. Tag sequences were then subcloned into pDrive-*KIP1* and pDrive-*CIN8*.

In order to overexpress Kip1-myc_12_ and Cin8-myc_12_ in yeast for subsequent purification and use in *in vitro* phosphorylation assays, *KIP1-12MYC* and *CIN8-12MYC* were subcloned into the BamHI site of the episomal vector YEpLGAL (a gift from Peter Kaiser), under the control of the *GAL1* promoter. YEpUGAL was generated by replacing the AatII-NarI segment of YEpLGAL containing the *LEU2* gene with the AatII-NarI segment containing the *URA3* gene from YEp195 [3].

To generate a pRS300 series integrating plasmid [4] with a *HIS2* marker that could be used for integration into the BF264-15DU wild-type yeast strain, the *HIS2* marker of YIpGAP2 was first amplified by PCR and TA cloned into pGEM-T-Easy (Promega). The *URA3* marker in pRS306 [4] was disrupted by digestion with NsiI and NcoI; the *HIS2* marker was cut from pGEM-T-Easy-*HIS2* with NsiI and NcoI, then ligated to the remainder of the pRS306 plasmid to yield pRS306H2.

To generate *KIP1* and *CIN8* alleles under the control of their own promoters, the respective 5’ and 3’ untranslated regions of the two genes were amplified by PCR from yeast genomic DNA and subcloned into pDrive-*KIP1* and pDrive-*CIN8*, thus resulting in pDrive-*KIP1*(native) and pDrive-*CIN8*(native). Constructs with C-terminal mCherry tags were placed under the control of their native promoters by subcloning only the 5’ untranslated region.

In order to integrate C-terminally tagged *KIP1* and *CIN8* alleles into the genome, they were subcloned into pRS305T and pRS306H2T, respectively, on KpnI and XbaI ends. The additional ‘T’ designation indicates the presence of the *ADH1* terminator sequence; *T_ADH1_* was PCR amplified from pFA6a-GFP(S65T)-*TRP1* [5] and TA cloned into pDrive, then excised with XbaI and SpeI for ligation to pRS305 and pRS306H2 cut with XbaI. Untagged ‘native’ *KIP1* and *CIN8* alleles were subcloned into pRS306 in order to integrate them into the genome.

p*GAL-SIC1*Δ*3P::HIS2* was constructed first by excising part of the *URA3* marker in pGAL-*SIC1Δ3P* [6] with a complete NsiI and partial NcoI digest. The *HIS2* marker was then cut from pGEM-T-Easy-*HIS2* with NsiI and NcoI, then ligated to the remainder of the plasmid.

p*ZSPC42-GFP* was constructed by digesting pIA29 [7] with EcoRI and SalI, then ligating to the *ble* gene, which encodes resistance to zeocin, excised with the same enzymes from pUC4KIXX (Pharmacia). p*NSPC42-GFP* was constructed by excising the *ble* gene from p*ZSPC42-GFP* using *EcoRI* and *SalI*, then ligating the remainder of the plasmid to the *natMX4* (nourseothricin resistance) cassette cut from pAG25 [8] using the same restriction enzymes.

**Strain construction.** For PCR-mediated deletions of *KIP1* and *CIN8*, primers with flanking regions homologous to either gene were used to amplify the following marker genes or cassettes from the respective plasmids: *LEU2* from pRS305 [4], *kanMX4* (kanamycin resistance) from pRS400 [9], and *hphMX4* (hygromycin B resistance) from pAG32 [8].

Disruption of *SIC1* with *URA3* was achieved by integrating the plasmid pTN27-8 (similar to pTNb18 used to disrupt *SIC1* with *HIS3*, described in [10]) after digestion with EcoRI and HindIII.

For tagging *KIP1* and *CIN8* at their endogenous locus by PCR, tagged *KIP1* and *CIN8* constructs were subcloned into the BamHI site of pK*CUP1* [11]. Either Pfu DNA polymerase (Stratagene) or KOD HotStart DNA polymerase (Novagen) was then used to amplify about 1kb of the respective gene sequence, the tag sequence, and the *kanMX2* marker in the plasmid for transforming yeast.

In order to label spindle pole bodies (SPBs) with Spc42-GFP, yeast strains not derived from SBY408 [12] were transformed with either pIA29 [7], p*ZSPC42-GFP* or p*NSPC42-GFP* linearized with Bsu361. To label microtubules with CFP-Tub1 (α-tubulin), yeast strains were transformed with pCFP-TUB1 [13] cut with StuI.

Strains carrying the *P_GAL1_-SIC1*Δ*3P* transgene were generated by transforming yeast with p*GAL-SIC1*Δ*3P* [6] linearized with EcoRV or StuI, or with p*GAL-SIC1*Δ*3P::HIS2* linearized with either SnaBI or MfeI.

pRS305T plasmids containing mCherry-tagged *KIP1* alleles were linearized with AflII before transforming yeast; pRS306H2T plasmids containing mCherry-tagged *CIN8* alleles were linearized with Bsu361. Untagged *KIP1* and *CIN8* alleles cloned into pRS306 were linearized with StuI.

**Spot assay for proliferation at permissive temperature following arrest at restrictive temperature for *cdc4-3*(ts) *sic1*Δ cells.** This spot assay was an extension of the asynchronous temperature shift experiment described previously; *cdc4-3 SIC1* and *cdc4-3 sic1*Δ cells were grown to log phase at 24°C as described in Materials & Methods and then shifted to 37°C for up to 6 h. Cells were collected from both cultures before the shift, and also at 2, 4, and 6 h after the shift from the *cdc4-3 sic1*Δ culture. A 2 x 10^6^ cells/ml suspension was prepared in YEPD from cells collected at each timepoint was then prepared and diluted serially to make 2 x 10^5^ and 2 x 10^4^ cells/ml suspensions. Three microliters of each suspension was spotted on a YEPD plate and the plate was subsequently incubated at ambient room temperature (~22°C).

**Clb5/Cdc28 kinase purification.** Soluble Clb5/Cdc28 kinase was prepared in a similar manner to that described for Clb2/Cdc28, except that Clb5-TAP was overexpressed from a different episomal plasmid, p*GAL-CLB5-TAP* [14], but also together with Cdc28 in a *swe1*Δ yeast strain (SBY1352, a gift from Daniel Lew).

**Identification of candidate kinases for non-CDK phosphorylation sites assigned during mass spectrometric analysis.** Amino acid sequences for Kip1-myc_12_ and Cin8-myc_12_ were analyzed using KinasePhos [15] and KinasePhos 2.0 [16] employing the default parameters. The predicted phosphorylation sites (Ser/Thr) and candidate kinases were then compared with the phosphorylation sites actually identified during LC/MS/MS analysis. Whille the E-value (for KinasePhos) and the SVM score (for KinasePhos 2.0) were taken into consideration when deciding on the most likely kinase for each site, candidate kinases not found in the *S. cerevisiae* genome were automatically eliminated.

**Supporting References**

1. Hoyt MA, He L, Loo KK, Saunders WS (1992) Two *Saccharomyces cerevisiae* kinesin-related gene products required for mitotic spindle assembly. Journal of Cell Biology 118: 109-120.

2. Shaner NC, Campbell RE, Steinbach PA, NG. GB, Palmer AE, et al. (2004) Improved monomeric red, orange and yellow fluorescent proteins derived from *Discosoma* sp. red fluorescent protein. Nature Biotechnology 22: 1567-1572.

3. Gietz RD, Sugino A (1988) New yeast-*Escherichia coli* shuttle vectors constructed with in vitro mutagenized yeast genes lacking six-base pair restriction sites. Gene 74: 527-534.

4. Sikorski RS, Hieter P (1989) A system of shuttle vectors and yeast host strains designed for efficient manipulation of DNA in *Saccharomyces cerevisiae*. Genetics 122: 19-27.

5. Longtine MS, McKenzie III A, Demarini DJ, Shah NG, Wach A, et al. (1998) Additional modules for versatile and economical PCR-based gene deletion and modification in *Saccharomyces cerevisiae*. Yeast 14: 953-961.

6. Verma R, Annan RS, Huddleston MJ, Carr SA, Reynard G, et al. (1997) Phosphorylation of Sic1p by G_1_ Cdk required for its degradation and entry into S phase. Science 278: 455-460.

7. Adams IR, Kilmartin JV (1999) Localization of core spindle pole body (SPB) components during SPB duplication in Saccharomyces cerevisiae. Journal of Cell Biology 145: 809-823.

8. Goldstein AL, McCusker JH (1999) Three new dominant drug resistance cassettes for gene disruption in *Saccharomyces cerevisiae*. Yeast 15: 1541-1553.

9. Brachmann CB, Davies A, Cost GJ, Caputo E, Li J, et al. (1998) Designer deletion strains derived from *Saccharomyces cerevisiae* S288C: a useful set of strains and plasmids for PCR-mediated gene disruption and other applications. Yeast 14: 115-132.

10. Nugroho TT, Mendenhall MD (1994) An inhibitor of yeast cyclin-dependent protein kinase plays an important role in ensuring the genomic integrity of daughter cells. Molecular and Cellular Biology 14: 3320-3328.

11. Haase SB, Winey M, Reed SI (2001) Multi-step control of spindle pole body duplication by cyclin-dependent kinase. Nature Cell Biology 3: 38-42.

12. Simmons Kovacs LA, Nelson CL, Haase SB (2008) Intrinsic and Cyclin-dependent Kinase-dependent Control of Spindle Pole Body Duplication in Budding Yeast. Molecular Biology of the Cell 19: 3243-3253.

13. Jensen S, Segal M, Clarke DJ, Reed SI (2001) A novel role of the budding yeast separin Esp1 in anaphase spindle elongation: evidence that proper spindle association of Esp1 is regulated by Pds1. Journal of Cell Biology 152: 27-40.

14. Ubersax JA, Woodbury EA, Quang PN, Paraz M, Blethrow JD, et al. (2003) Targets of the cyclin-dependent kinase Cdk1. Nature 425: 859-864.

15. Huang H-D, Lee T-Y, Tzeng S-W, Horng J-T (2005) KinasePhos: a web tool for identifying protein kinase-specific phosphorylation sites. Nucleic Acids Research: W226-229.

16. Wong Y-H, Lee T-Y, Liang H-K, Huang C-M, Wang T-Y, et al. (2007) KinasePhos 2.0: a web server for identifying protein kinase-specific phosphorylation sites based on sequences and coupling patterns. Nucleic Acids Research: W588-594.
